# Cover crop-driven shifts in soil microbial communities could modulate early tomato biomass via plant-soil feedbacks

**DOI:** 10.1038/s41598-022-11845-x

**Published:** 2022-06-01

**Authors:** Micaela Tosi, John Drummelsmith, Dasiel Obregón, Inderjot Chahal, Laura L. Van Eerd, Kari E. Dunfield

**Affiliations:** 1 School of Environmental Sciences, University of Guelph, 50 Stone Rd. E, Guelph, ON N1G 2W1 Canada; 2 School of Environmental Sciences, University of Guelph, Ridgetown Campus, Ridgetown, ON N0P 2C0 Canada

**Keywords:** Soil microbiology, Microbial ecology, Agroecology, Biodiversity

## Abstract

Sustainable agricultural practices such as cover crops (CCs) and residue retention are increasingly applied to counteract detrimental consequences on natural resources. Since agriculture affects soil properties partly via microbial communities, it is critical to understand how these respond to different management practices. Our study analyzed five CC treatments (oat, rye, radish, rye-radish mixture and no-CC) and two crop residue managements (retention/R+ or removal/R−) in an 8-year diverse horticultural crop rotation trial from ON, Canada. CC effects were small but stronger than those of residue management. Radish-based CCs tended to be the most beneficial for both microbial abundance and richness, yet detrimental for fungal evenness. CC species, in particular radish, also shaped fungal and, to a lesser extent, prokaryotic community composition. Crop residues modulated CC effects on bacterial abundance and fungal evenness (i.e., more sensitive in R− than R+), as well as microbial taxa. Several microbial structure features (e.g., composition, taxa within Actinobacteria, Firmicutes and Ascomycota), some affected by CCs, were correlated with early biomass production of the following tomato crop. Our study suggests that, whereas mid-term CC effects were small, they need to be better understood as they could be influencing cash crop productivity via plant-soil feedbacks.

## Introduction

Cover crops (CCs) increase the number of plant species in crop rotation (i.e., diversification), extend the time under living plant cover (i.e., ‘perennialization’ of annual cropping systems), and can act as mulch or green manure. These aspects aim to resemble some aspects of natural ecosystems such as their higher diversity, complexity and functionality, which make them less reliant on external inputs^1^. Thus, it is not surprising that CCs can provide benefits such as increasing soil organic carbon and nutrients, improving soil structure, minimizing soil erosion and suppressing weeds^2–4^. Under certain conditions, CCs may also increase crop yields and productivity^5–7^.

These changes in soil properties and plant growth associated with CCs occur mainly via changes in the soil biota^8^. For instance, CCs were shown to shift soil microbial community composition^9–11^, and increase microbial abundance and diversity^3,12,13^. They may also have functional repercussions by favoring or affecting specific microbial groups or life strategies. For example, depending on the plant species, CCs were shown to promote cellulolytic, nitrogen-fixing, disease-suppressive and ruderal bacteria, saprotrophic and endophytic fungi, and to shift arbuscular mycorrhizal fungi (AMF) composition^9,14–16^. The influence of CC species composition on soil microbial communities is not yet fully clear, but different species could favor or inhibit specific microbial groups^11,17,18^. Yet, in some studies, CC species did not affect soil microbial biomass, composition or functional traits markedly^18^, or their effect was surpassed by other CC management strategies^19^.

At the same time, retaining crop residues can provide physical protection to soils, while also constituting a source of carbon and nutrients for soil biota^20,21^. Crop residue retention was shown to increase microbial biomass and activity^20,21^, as well as culturable catabolic diversity^22^. Controls over microbial diversity are less clear, although changes in community composition were observed when residues were removed^23–25^.

Despite the available evidence, the nature of soil biological processes is complex and results are dependent on site-specific factors such as soil type, climate and topology^2,12,13,20,21^. Additionally, interactions between different agricultural practices seem to be crucial in determining the response of soil properties and crop productivity^5,18,26^. For example, if crop residues need to be removed for agricultural reasons^26,27^, CCs could alleviate some of the negative consequences of this practice on soil health^28^.

Previous studies in this 8-year horticultural crop rotation suggested that repeated CC utilization could improve soil health and crop growth^6,26,27^. The goal of this study was to better understand the microbial mechanisms underlying those changes by studying the structure of soil microbial communities. Besides a no-CC control, CC treatments consisted of oat (*Avena sativa* L.), rye (*Secale cereale* L.), radish (*Raphanus sativus* L.), and a mixture of the last two species, which present distinct root structures and nutrient requirements^29^. While most microbiome studies in the literature focus on prokaryotes and short-term responses to CC (i.e., single growing season), this experiment evaluates both prokaryotes and fungi in the medium term (i.e., 6 times in 8 years). Soil microbial community structure was studied using quantitative PCR (qPCR) to assess abundance, and high-throughput sequencing to assess diversity and community composition. These effects were evaluated in interaction with the removal (R−) or retention (R+) of winter wheat crop residues.

We hypothesized that both the inclusion of CCs (i.e., CC vs. no-CC) and residue retention would shift microbial community composition and increase microbial abundance, possibly via changes in the belowground environment (e.g., C and nutrient inputs, moisture, temperature fluctuations). In addition, CC species with different morphological and functional traits would promote different microbial groups, leading to changes in community composition and potentially increasing alpha diversity. For all variables, we expected to find some degree of interaction between CCs and crop residue management, since they both act as sources of C and nutrients, and both can modify the soil physical environment. Finally, even though soil microbial communities undergo seasonal fluctuations, we expected some degree of relationship between microbial structure in the fall and cash crop growth in the following spring (i.e., plant–soil feedbacks). In particular, specific taxa or functional groups present in the fall could have a role in soil functions or plant–microbe interactions the following crop season.

## Results

### Response of soil prokaryotic communities to CC and residue treatments

**Figure 1:**
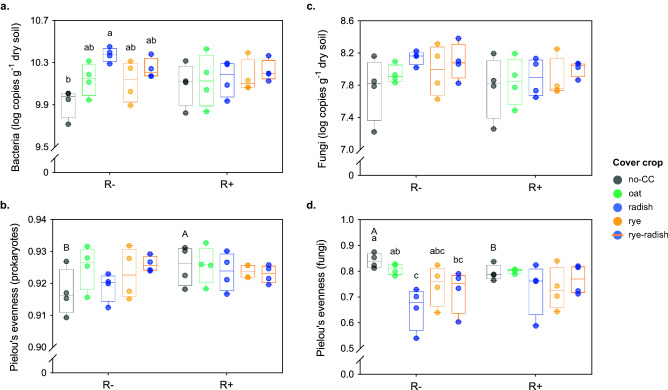
Microbial community structure in soils under different CCs and residue management: (**a**, **c**) Bacterial and fungal abundance, respectively; (**b**, **d**) Prokaryotic and fungal ASV evenness (Pielou’s index), respectively. Colors indicate different CCs and R+/R− refers to main crop residues present or absent, respectively. Different lowercase letters show significant differences between CCs within residue treatments, while different uppercase letters show differences between residue treatments (Tukey test, alpha = 0.05). Letters are only shown for comparisons with P < 0.05. R−/R+ refers to main crop residues absent or present, respectively.

CCs affected bacterial abundance only in R-, where 16S rRNA copy numbers in no-CC were lower than radish and marginally lower than rye-radish (P = 0.005 and P = 0.051, respectively) (P = 0.005 and P = 0.051, respectively) (Fig. 1a). Intermediate values were found for cereal CCs (oat and rye) (Fig. 1a). Prokaryotic alpha diversity was mostly unaffected by CC and residue management, with the exception of no-CC soils having higher evenness in R+ than R− (Fig. 1b, Table S1). Still, Venn diagrams showed that rye-radish, followed by rye, was the CC treatment with the highest number of total and unique ASVs (Fig. S1a).

**Figure 2:**
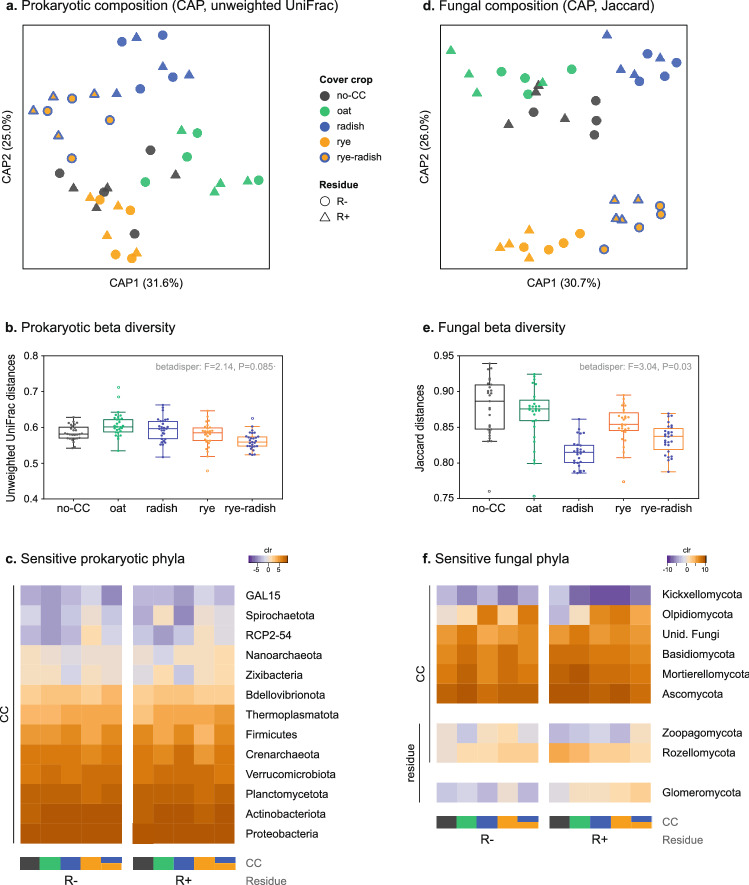
Microbial community structure in soils under different CCs and residue management: (**a**, **d**) Canonical analysis of principal coordinates (CAP) showing effect of CCs (and residue, for fungi); (**b**, **e**) Beta diversity as dispersion between different plots within each CC treatment, including betadisper results; (**c**, **f**) Heatmaps showing changes in the relative abundance of microbial phyla. In heatmaps, color gradient represents average relative abundance expressed as centered log-ratio (clr), and CC treatments are indicated by colored bars. R−/R+ refers to main crop residues absent or present, respectively. Heatmaps were created with R package ‘gplots’ (github.com/talgalili/gplots).

The phylogenetic composition of prokaryotic communities was not affected by CC or residue management according to PERMANOVA (Table S2), although some CC effects were evident in the partial CAP (Fig. 2a). The largest compositional differences were observed between oat and rye-radish (CAP1 = 31.6%), followed by rye *vs.* radish (CAP2 = 25.0%) (Fig. 2a). CAP2 displayed the following gradient between those two CCs: rye < no-CC/oat < rye-radish < radish (Fig. 2a). Oat communities also presented higher beta diversity than rye-radish (Fig. 2b). Still, soil prokaryotic communities presented high unexplained variability (unconstrained inertia = 74.18%) and high dispersion within CC treatments (Fig. 2b).

**Figure 3:**
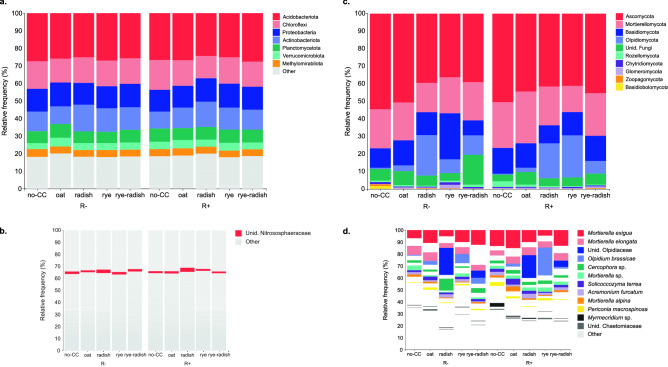
Changes in the relative frequency (%) of soil microbial taxa in different CCs and residue management treatments: (**a**, **c**) Prokaryotic and fungal phyla, respectively; (**b**, **d**) Prokaryotic and fungal species (or highest classification available) with visually evident changes in frequency, respectively. Each treatment is represented by pooled reads from four field replicates. In (**a**), all phyla in the legend are bacterial and lower abundance phyla were pooled as “Other”. In (**b**, **d**), taxa with no visually clear changes in frequency were pooled as “Other”. R−/R+ refers to main crop residues absent or present, respectively.

Cover crops also changed the relative abundance of some prokaryotic taxa. In terms of bacterial phyla, radish favored Actinobacteriota more than other CCs and rye promoted RCP2-54 (Fig. 2c). Especially in R+, cereal CCs were detrimental to Firmicutes (Fig. 2c). Among archaea, Thermoplasmatota was lower in no-CC and Crenarchaeota was higher in radish. The response of some phyla was modulated by residue management (e.g., Crenarchaeota, Verrucomicrobiota, GAL15, Spirochaetota, Nanoarchaeaeota and Zixibacteria) (Fig. 2c). Even phyla with a more consistent response between residue managements (e.g., RCP2-54, Firmicutes) were generally more sensitive in R+ than R− (Fig. 2c). At the genus level, indicator species analysis identified 37 bacterial and 2 archaeal taxa associated with specific CCs, although half of them were detected in either R− or R+ (Table S3). Overall, oat and radish had less indicator taxa while the opposite was observed for rye-radish (11 and 16 *vs.* 27, respectively) (Table S3). Besides, ALDEx detected a large number of taxa associated to CCs, among which 12 were also indicator species (Table S4). One of these was an unidentified Nitrososphaeraceae archaeon with higher relative frequency (%) in radish soils (Fig. 3b). Besides this, and a slightly higher frequency of Actinobacteria and lower Acidobacteria in radish, treatments presented similar proportions of different prokaryotic taxa (Fig. 3a,b).

### Response of soil fungal communities to CC and residue treatments

The response of soil fungal communities to CC and residue treatments often contrasted that of prokaryotes. Fungal abundance was not affected by CC or residue management (Fig. 1c). Even though fungal richness was not sensitive to the applied treatments (Table S1), radish-based CCs presented the highest number of total and unique ASVs (Fig. S1b). Fungal evenness was affected by CCs only in R−, where no-CC was higher than radish (P = 0.011) and rye-radish (P = 0.049), and oat higher than radish (P = 0.011) (Fig. 1d). Also, no-CC presented higher fungal evenness in R− than R+ (P = 0.029) (Fig. 1d).

Fungal community composition was affected by CCs (R^2^ = 0.22, P = 0.042) but not by residue management (PERMANOVA, Table S2). CAP indicated that cc-based differences were mostly explained by the presence of radish (CAP1 = 23.0%), followed by rye (CAP2 = 22.0%) (Fig. 2d). CAP1 showed not only a CC gradient (oat < rye < no-CC < rye-radish < radish), but also a slight distinction between R+ and R− (Fig. 2d). The stronger influence of radish and rye on fungal composition was also evident in the similarities of rye-radish communities to each individual CC species (Fig. 2d), as well as the lower beta diversity of radish, rye-radish and rye (Fig. 2e). Despite these effects, fungal community composition also exhibited a large proportion of unexplained variability (unconstrained inertia = 76.91%) and relatively high dispersion within CC treatments (Fig. 2e).

Both CC and residue treatments affected the relative abundance of fungal taxa, again with interactions between them. Oat and no-CC increased Mortierellomycota and Ascomycota, and, in R+, Basidiomycota (Fig. 2f). Oat also favored unidentified fungi while, in R−, both cereals increased Basidiomycota and reduced Kickxellomycota (Fig. 2f). In R+, Kickxellomycota was negatively affected by all CC species compared to no-CC (Fig. 2f). Olpidiomycota, one of the most sensitive phyla, was lower in no-CC, followed by both cereals in R− and only by oat in R+. Indicator species analysis found 20 genera associated with the different CC treatments, half of them in either one of the two residue treatments (Table S5). Similar to prokaryotes, oat presented the lowest number of indicator taxa (5 out of 20) (Table S5). Besides, ALDEx detected 24 fungal taxa associated to CCs, 5 of which were also indicator species (Table S6). As opposed to prokaryotes, changes in the relative frequency of fungal taxa were clearer (e.g., higher Olpidiomycota and lower Ascomycota in radish, rye and rye-radish) (Fig. 3c). Radish, which had the lowest fungal evenness, presented a high frequency of an unidentified Olpidiaceae (~ 21%) and, especially in R−, *Cercophora* sp. (~ 10%) (Fig. 3d). This Olpidiaceae fungus, absent in other CC treatments, was an indicator species for radish (Fig. 3d, Table S5). Contrarily, cereals, but mostly rye, presented higher proportion of *Olpidium brassicae* (rye R+: 23.47%, rye R−: 7.58%) (Fig. 3d).

Retaining crop residues (R+) increased the relative abundance of Glomeromycota and Rozellomycota, as well as lower Zoopagomycota, with some variation between CC treatments (Fig. 2f). *Mortierella* was a highly abundant genus in most samples (14.6–29.4%), and, except for rye, it was more dominant in R+ than R− (Fig. 3d). In no-CC, other taxa were also more dominant in R+ than R−, consistently with the lower evenness: Rozellomycota (3.19% vs*.* 0.80%), *Myrmecridium* sp. (3.02% vs. 0.19%), *Periconia macrospinosa* (3.2% vs. 0.47%), *Solicoccozyma terrea* (2.61% vs. 1.65%), *Acremonium furcatum* (3.00% vs. 1.97%), and unidentified Chaetomiaceae (0.62% vs. 0.17%) (Fig. 3c,d).

### Relationship between soil microbial communities and plant growth

**Figure 4:**
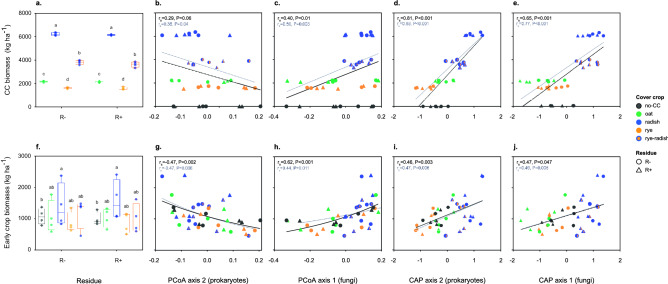
Cover crop (CC) and early tomato aboveground biomass under different CCs and residue management treatments, and relationship between these variables and microbial community composition: (**a**) Response of CC biomass; (**b**, **c**) Relationship between CC biomass and PCoA axes from prokaryotic and fungal data, respectively; (**d**, **e**) Relationship between CC biomass and CAP axes from prokaryotic and fungal data, respectively; (**f**) Response of early tomato crop biomass; (**g**, **h**) Relationship between early crop biomass and PCoA axes from prokaryotic and fungal data, respectively; (**i**, **j**) Relationship between early crop biomass and CAP axes from prokaryotic and fungal data, respectively. In (**a**, **f**), different letters show significant differences between CCs for each residue treatment (Tukey test, alpha = 0.05). In (**b**–**e**) and (**g**–**j**), Spearman correlations (rs) and trendlines were calculated both including and excluding no-CC (black and grey values/lines, respectively). PCoA and CAP carried out with unweighted UniFrac and Jaccard distances for prokaryotic and fungal communities, respectively. Eigenvalues for prokaryotes PCoA: Axis 1 = 0.094, Axis2 = 0.057; eigenvalues for fungi PCoA: Axis 1 = 0.078, Axis2 = 0.055. CAP results shown in Fig. 2. Symbol colors indicate different CC treatments and R−/R+ refers to main crop residues absent or present, respectively.

At the time of sampling (October 2015), CCs presented different levels of biomass, as follows: rye < oat < rye-radish < radish (P < 0.001) (Fig. 4a). Cc biomass was positively correlated with soil bacterial abundance (Spearman r (r_s_) = 0.50, P = 0.001) (Fig. S2a) and, to a lesser extent, with fungal abundance (r_s_ = 0.30, P = 0.037) (Fig. S2b). In addition, higher CC biomass was associated with higher fungal ASV richness (r_s_ = 0.39, P = 0.014) and lower fungal evenness (r_s_ = -0.46, P = 0.003) (Fig. S2b). Correlations were also found between CC biomass and microbial community composition (PCoA axes), especially for the fraction of variance explained by CC treatments (CAP axes) (Fig. 4b-e). In both cases, correlations were detected with the PCoA/CAP axes that distinguished radish-based from other CCs (e.g., Fig. 2a,d). In the case of fungi, when excluding no-CC, a positive correlation was also found between CC biomass and CAP2 (r_s_ = 0.51, P = 0.003). Correlation values were always stronger when excluding no-CC, which we considered as having no aboveground biomass at sampling (Fig. 4b-e).

We also looked for a relationship between soil microbial community data and early crop biomass of the following’s year tomato crop, after transplant (July 2016). Early crop biomass was higher with radish than no-CC (P = 0.002), particularly in R+, with no clear differences among other CCs (Fig. 4f). There was some degree of correlation between early crop biomass and CC biomass (r_s_ = 0.30, P = 0.06), especially when excluding no-CC (r_s_ = 0.40, P = 0.02). Early crop biomass was also positively correlated with fungal abundance (r_s_ = 0.35, P = 0.026) and ASV richness (r_s_ = 0.47, P = 0.002) (Fig. S2b), and marginally with bacterial abundance (Pearson r = 0.29, P = 0.06) (Fig. S2a). Microbial community composition, both unconstrained (PCoA axes) and constrained (CAP axes), was also associated with early tomato growth (Fig. 4g-j). These correlations were found for the same axes as the ones correlated with CC biomass, as well as fungal CAP2 both including (r_s_ = 0.33, P = 0.036) and excluding no-CC (r_s_ = 0.38, P = 0.034). In all correlations with early crop biomass, no major changes were observed when including/excluding no-CC from the analysis (Fig. 4g–j).

**Figure 5:**
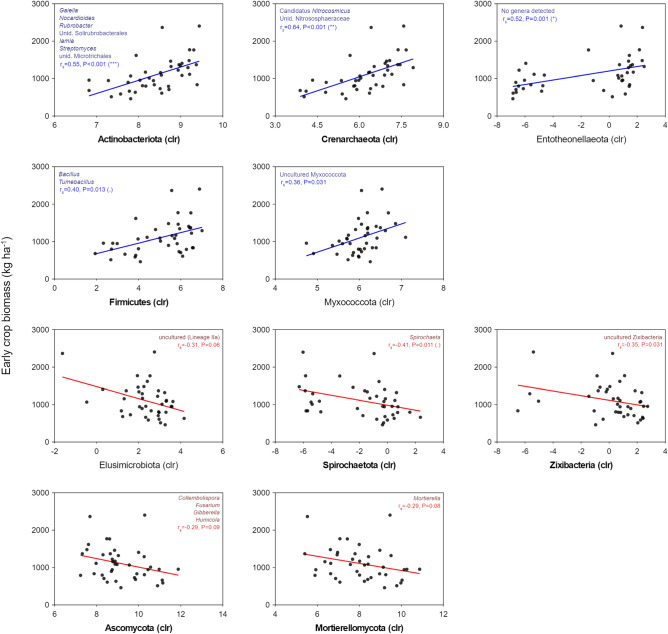
Soil prokaryotic and fungal phyla positively (blue) and negatively (red) correlated with early tomato growth in the following year. In each plot, Spearman correlation coefficients (r_s_) and P values from ALDEx (*aldex.corr*) are shown. If applicable, genera correlated to early crop growth are shown within each phylum’s plot (see Table S7 for full id.). Phyla names in bold were also sensitive to CCs (see Fig. 2c,f). Symbols between brackets represent Benjamini–Hochberg corrected P values, when significant: ***< 0.001, **< 0.01, *< 0.05, < 0.10.

Finally, we explored which taxa, among those correlated with early crop growth, were also sensitive to CC treatments. For example, Actinobacteriota, Crenarchaeota and Firmicutes were positively correlated with early crop biomass, while the opposite relationship was found for Spirochaetota and Zixibacteria (Fig. 5). At the genus level, 36 prokaryotic taxa were both sensitive to CCs and correlated with tomato growth (20 negatively and 16 positively) (Fig. S3a, Table S7). More than half of these taxa belonged to 4 out of 16 phyla: Actinobacteria (8), Proteobacteria (4), Chloroflexi (4) and Acidobacteria (4) (Table S7). Besides, only 12 of them belonged to phyla also associated with early crop growth (Fig. 5). Notably, except for *Actinocorallia*, Actinobacteria showed a positive relationship with crop growth (e.g., *Nocardioides*, *Iamia*, *Streptomyces, Gaiella* and *Rubrobacter*) (Fig. 5, Table S7). Contrarily, all Acidobacteria and three out of 4 Chloroflexi, most of them uncultured, were negatively associated with early tomato growth (Fig. 5, Table S7). Within the archaeal phylum Crenarchaeota, members of the family Nitrososphaeraceae showed a positive correlation with crop growth, while the opposite was found for a member of Nitrosopumilaceae (Fig. 5, Table S7). In fungal communities, 2 phyla (Ascomycota and Mortierellomycota) and 4 genera within these phyla were negatively related to early crop biomass while also being affected by CCs (Fig. 5, Fig. S4b, Table S7). Three of these taxa belonged to the phylum Ascomycota (*Fusarium*, *Collembolispora*, *Humicola*) and the fourth one, *Mortierella*, to Mortierellomycota. Overall, these prokaryotic and fungal genera were relatively abundant (clr > 0) and, as expected, those positively associated with crop growth were generally higher in radish or radish-based CCs and vice versa (Fig. S3, Table S7).

## Discussion

Cover crops can shape the soil microbiome by providing different C and nutrient sources via rhizodeposits and litter, via signaling or allelopathic compounds in root exudates (directly or via other organisms), or by modifying the soil abiotic environment^13,30^. In this study, CCs affected some aspects of soil microbial structure, consistently with other soil biological, chemical and physical properties^27^. Still, short- and mid-term microbial responses were relatively minor considering CCs had been applied for 7 successive years and were also present at sampling. Some methodological aspects may have had a role in minimizing the detectable impacts of CC treatments. For instance, plant-driven effects may have been more evident in the root vicinity (e.g., rhizoplane and rhizosphere) than the bulk soil^31^. Furthermore, in a diverse crop rotation like this one (i.e., legumes, cereals, nightshade plants and cucurbits), microbial communities could be more adapted to frequent shifts in plant species^32^ and less responsive than those from less diverse rotations. On the other hand, the CC species we tested may also have played a role, as CCs with legumes or diverse mixtures may have exerted stronger structural changes in soil microbes^3,13,33^. Finally, because microbial structure and response can fluctuate with the season^26,34^, or as CCs grow and their residues decompose^14,19^, greater changes may have been detected at another time point.

Other management-related and site-specific factors also likely influenced the microbial response observed^9,10,15,35^. Relatively high initial organic matter levels (Table S8) may have hindered the microbial response, as baseline microbial abundance and diversity levels could have also been high. Chemical CC termination could have negatively impacted plants and soil microorganisms lessening microbial population responses^12^. Likewise, small effects could be attributed to conventional tillage^9,13^, although a meta-analysis by Kim et al*.*^12^ found the opposite, probably because CCs mitigate the detrimental effects from tillage.

In general, CC effects were driven by plant species identity more so than by CC inclusion or increased plant richness in the rotation. The rye-radish mixture sometimes produced unique results that did not resemble the effect of either of the individual species (e.g., relative abundance of taxa) or the combination of both (e.g., fungal community composition). Yet, it did not enhance the response of abundance or diversity compared to each individual species. Radish, followed by rye, was the CC species exerting the strongest effects on microbial structure. Consistently, radish had higher aboveground biomass at sampling, it can produce antifungal compounds^13^, and it has a distinct root morphology and nutrient requirements compared to cereals^29^. Substrate input quality seemed to be less influential than quantity, considering the aboveground biomass C:N ratio was relatively stable^6^, although belowground inputs may have been more relevant in the case of fall CCs^4,16^*.* Besides, higher input quality could be expected on radish inputs, as it tended to have higher N content^6^ and usually has higher P content^36^ than cereal inputs. The influence of rye CCs, especially on fungi may explained by the production of allelochemicals. These compounds are mainly phytotoxins, some of which can be metabolized by specialized soil fungi and other microorganisms^37,38^. Rye also had the lowest aboveground N content and N uptake^26^, possibly leading to lower C:N inputs, and it was the only CC that overwintered, hence exposing soils to living roots for longer periods.

Soil prokaryotic and fungal communities responded quite differently to CCs. While bacteria were more sensitive in terms of abundance, fungi showed higher sensitivity in terms of diversity and composition. Both bacterial and fungal biomass and abundance could be enhanced by the increased plant cover and richness of CCs^9,10,12,18,33,39^, sometimes depending on the CC species^18,40,41^. Here, bacterial abundance seemed to have responded to the overall C supply^9,13^, as it behaved similarly to aboveground biomass (i.e., higher in radish)^17,39^ and was only sensitive in R−. Contrarily, fungal abundance was not responsive to CCs, contrarily to previous findings^42^. Considering soil fungi are as able as bacteria to utilize rhizodeposits^43^, we hypothesize their lack of response is the product of tillage damaging hyphae and other fungal structures. Technical biases from DNA extraction and qPCR could also be involved in this response^44^.

Microbial community composition can be shaped by plant species identity and functional traits^45,46^, including via CCs^9–11,34^. The observation that fungi responded in a more qualitative way (i.e., composition, diversity) than prokaryotes could have many reasons. Firstly, the lower overall diversity of fungi could have made small compositional changes more evident. Here, shifts in community composition were not only small but mostly in terms of presence-absence, which give relatively more importance to rarer organisms^47^. On the other hand, fungi may have been highly sensitive to radish antifungal compounds and rye phytotoxins. In fact, fungal composition was mainly driven by the presence of these two species, while prokaryotic community composition was mostly affected by oat. Radish also reduced fungal evenness by disproportionally promoting some fungi over others (e.g., unidentified Olpidiaceae and *Cercophora* sp.). While Olpidiaceae fungi are potential brassica pathogens^48^, saprotrophs like *Cercophora* may have preference for specific radish inputs. This hypothesis is supported by changes in evenness (and *Cercophora* frequency) being less clear in R+, where wheat residues acted as an alternative C source. It is also possible that high C:N wheat residues caused N immobilization in R+, leading to lower N availability and cascading effects on soil microbiota. Besides this, overall effects on microbial alpha diversity were negligible. We had predicted alpha diversity could be enhanced by the increased plant and resource diversity of CC treatments^49^, but this was observed only as a trend in rye-radish soils. Consistently, clear positive effects of CC on microbial diversity are limited^10^ or small^12^, and CCs adding only one or two extra species may also minimize their impact to more diverse CC mixtures^32^.

Microbial shifts were also reflected in the relative abundance of specific phyla and genera, but our results did not resemble the changes in functional groups found by other authors^9,14–16^. For both prokaryotes and fungi, the response of different taxa was strongly affected by the residue management in place. The functional relevance of sensitive prokaryotic taxa remains unknown for being uncultured and/or partially identified. In spite of its antifungal activity^13^, radish promoted two potential fungal pathogens of brassica plants: Olpidiaceae and *Leptosphaeria*^48^. With cereals, especially rye, unidentified Olpidiaceae was replaced by *O. brassicae*, an obligate plant pathogen commonly found in brassicas that can survive in bulk soil as spores^48^. Although this result seems contradictory, the unidentified Olpidiaceae could in fact be *O. brassicae*, while the *O. brassicae* in rye could either be a different genotype^50^ or *O. virulentus*, a related species more frequently found in non-brassicas^48^. Neither *Olpidium* nor *Leptosphaeria*, however, are likely pathogens of tomato, which may explain why radish was still the treatment with highest early crop growth. The symbiotic Glomeromycota fungi were not markedly affected by radish, despite brassicas not associating symbiotically with AMF and sometimes affecting their populations^13^. Most probably, main crops in the rotation kept soil AMF populations stable or, contrarily, there was an overall negative effect of conventional tillage.

Our results also revealed residue management could modulate the response of soil microbial communities (e.g., bacterial abundance, fungal evenness, microbial taxa) to CCs and vice versa. Since residue management was not associated with aboveground CC biomass, we suggest such modulation did not occur via plant growth. On the contrary, it seemed to be explained by the fact that both CCs and main crop residues act as C and nutrient sources for the soil biota, as previously reported^51^. For example, detrimental effects of no-CC and oat on soil bacterial abundance were buffered when retaining crop residues (R+), probably by providing an additional C and nutrient source. A similar buffering effect was observed on fungal evenness, as CC effects were smaller in R+ than R−. Wheat residues reduced evenness in no-CC, as discussed above, but they also increased it in radish-based treatments, perhaps by acting as an alternative substrate for a wider range of fungi.

Besides constituting a source of C and nutrients, residues also modify soil temperature and moisture content^21^, therefore modifying the soil environment. Even so, consistently with previous results on other soil properties^27^, residue management effects in this study were smaller than CC effects, and negligible for most variables. Throughout the 8-year trial, crop residues were removed only twice while CCs were applied 6 times, but we expected clearer effects because it was carried out on the sampling year. Likely, crop residues represent a more limited source of C and nutrients, while growing CCs provide multiple sources (e.g., aboveground residues, belowground residue, rhizodeposits). This is supported by previous findings highlighting the importance of belowground inputs of CCs as a C source for soil microorganisms^33,52^.

In spite of residues being incorporated via tillage ~ 2 months before sampling, microbial abundance did not increase in R+. We might have missed a transient response^53^ or, alternatively, tillage accelerated residue mineralization and a large proportion of C was lost as CO_2_ instead of being used for microbial growth^21^. The latter is supported by higher C mineralization levels one month before sampling^27^. In terms of composition and taxa, crop residue affected fungi more so than prokaryotes, possibly because fungi are more capable of breaking down plant cell wall polymers. Surprisingly, residue retention affected three phyla hosting few or no saprophytic taxa: it favored Glomeromycota (AMF, obligate symbionts) and Rozellomycota (mostly animal and protist parasites), while reducing Zoopagomycota (animal, protist and mycoparasite, sometimes saprophytic)^54^. Such effects may have been indirect, via other soil organisms, standing CCs and/or previous crops. Recent studies also suggest AMF could remain viable after host shoots are removed^55^ and even participate in organic matter decay^56^.

In fallow soils (no-CC), retaining crop residues (R+) had contrasting effects on prokaryotic and fungal evenness. Since prokaryotic evenness increased together with bacterial abundance, it seems a wide array of bacterial taxa by the overall higher resource availability in no-CC R+. Contrarily, the incorporated wheat straw favored only a few soil fungi over others. Some of these fungi may have increased during the wheat crop and, where no other substrates were available (i.e., no-CC), they remained dominant in the retained residues. This could be the case of *Fusarium* sp. and *Periconia macrospinosa* (potential plant pathogens or endophytes), as well as *Mortierella exigua* (saprotrophic or root-associated).

Early growth of tomato crops after transplant can be critical benefit for crop establishment. Soil microbial communities can affect plant growth and productivity^30,57^ via changes in soil physicochemical properties or direct/indirect interactions. In our study, early tomato growth was associated with soil microbial composition and specific microbial taxa (affected by cc), as well as fungal abundance and richness (not affected by cc). Higher fungal abundance could promote plant growth via enhanced organic matter cycling and aggregation, while higher fungal diversity could be associated with increased functional capacity and disease suppression. On the other hand, shifts in the soil microbiome could alter the abundance of pathogens, plant-growth promoting organisms, or organisms involved in key nutrient transformations^57,58^. Here, fluctuations in soil borne pathogens could be linked to tomato growth, considering its negative correlation with two known pathogenic fungi: *Fusarium* (*F. solani* and *Fusarium* sp.) and *Gibberella* (*G. intricans*)^54^. While *Fusarium* sp. could have infected tomato crops directly, *F. solani* and *G. intricans* are not common tomato pathogens but may be compromising the growth of other crops in the rotation. Unexpectedly, *Mortierella* was also negatively correlated with crop growth despite bearing several PGP strains, some of them associated with *Fusarium* wilt disease suppression^59^. Yet, the latter could explain why it is sometimes correlated with *Fusarium*^60^. Contrarily, bacterial taxa with potential PGP and/or disease-suppressive traits positively correlated with early crop growth, including the phylum Firmicutes (*Bacillus* and *Tumebacillus*)^61^ and Actinobacteria (*Iamia*, *Nocardioides*, *Streptomyces*, *Gaiella*)^58,62^. In terms nutrient cycling regulation, two ammonia-oxidizers (AOA) from Nitrososphaeraceae positively correlated with crop growth, although the opposite was found for two other AOA taxa (*Candidatus Nitrosotenuis*, Nitrosopumilaceae). This result could reflect different ecological adaptations or substrate affinity between AOA taxa^63^.

Microbial changes associated with both CC treatments and early tomato growth could point out to the existence of plant-soil feedbacks. In previous studies, CCs led to shifts in the bulk or rhizosphere microbiome of cash crops^33,42,64^, which could potentially affect crop health and fitness^58,65^. Here, early tomato growth was highest under radish-based CCs, which were the ones also favoring fungal abundance and diversity, as well as causing the strongest compositional shifts. Besides, radish-based CCs generally presented lower relative abundance of the potentially pathogenic fungi, as well as a higher relative abundance of potential PGP and disease-suppressive bacteria, as previously reported^13,15,66^. Still, we cannot discard the influence of abiotic factors, such as the higher mineral N of radish-cultivated soils in the following spring (Table S8). Radish residues, with their higher quantity and quality, may have been rapidly mineralized in the spring^7,36^, leading to higher N availability for tomato crops.

Overall, in this 8-year horticultural crop rotation, CCs had small effects on soil microbial community structure, despite their repeated application and being present at fall sampling. Still, these small effects evidenced belowground-aboveground interactions that could have productive implications. Even though these results are exploratory, the fact that there was some degree of relationship between cc-associated microbial communities (fall 2015) and early crop growth in the following season (spring 2016) suggests the existence of plant-soil feedbacks linking CCs and cash crops^1^. Detecting such links in field trials constitutes a valuable data resource for future studies where these hypotheses are put to the test. Such studies should not disregard the influence of site-specific conditions and agricultural management^12,67^, especially those practices affecting C and nutrient inputs or imposing a significant disturbance on the soil microbiome left by the CCs^14,67,68^.

## Methods

### Site description

The experimental site was located at the Ontario Crops Research Centre, Ridgetown, Ontario, Canada (42.44° N, 81.88° W, 201 m.a.s.l). The soil at the site is Orthic Humic Gleysol with a sandy loam texture (68% sand, 21% silt, 11% clay)^27^. The trial (established 2008) consisted in a diverse horticulture and grain crop rotation comparing different CC species compositions and main crop residue management practices^26^ (Table S9). The experimental design was a split-plot randomized complete block design with four replicates, where CC was applied to whole plots and residue management to sub-plots of 6 m × 8 m^27^. The main crop was winter wheat (*Triticum aestivum* L.), which was harvested mechanically on 7 August 2015. Following harvest, aboveground residues were either removed or retained (R− and R+, respectively). In R−, straw was removed by cutting to ~ 10 cm from the soil surface and removing by hand with a rake, while in R+, wheat residues were uniformly distributed throughout the sub-plot. Besides a no-CC control, the trial comprised four different CCs: oat (*Avena sativa* L.), fall rye (*Secale cereale* L.), radish (*Raphanus sativus* L.) and an intercropped mixture of the last two species. These crops were planted on 17 August 2015, using a seed drill, at 81, 67, 16, and 9 + 34 kg ha^−1^, respectively^27^. Before planting CCs, a light tillage (disk followed by cultivator) was applied to all treatments in order to incorporate the residues in R+ and prepare the seed bed. Except for rye, CCs were terminated by winter-kill. To terminate rye and control weeds, the entire trial was sprayed with glyphosate (810 g a.e. ha^−1^) on early May 2016. Before transplanting tomato (*Solanum lycopersicum* L.) (late May 2016), CCs were incorporated in the soil using disk and two cultivator passes. More details about the trial, including the diverse crop rotation, can be found in Tables S9, S10 and previous publications^26,27^.

### Soil sampling and processing for microbial analyses

Bulk soil samples were collected on 19 October 2015, two months after planting CCs, which were in vegetative stage. In each sub-plot, we collected 9 soil cores (0–10 cm depth, 2.5 cm diameter) along two perpendicular transects, excluding ~ 1 m from the plot borders. Soil samples, collected aseptically (i.e., disinfecting tools with ethanol 70% W/V, placing soil in sterile bags), were manually homogenized, stored at 4 °C and processed within 24 h for DNA extraction. A sub-sample was used to measure gravimetric dry weight.

Soil DNA was extracted using a MO BIO PowerSoil DNA Isolation Kit (MOBIO Laboratories, Inc.) according to the manufacturer’s guidelines. DNA concentration and purity were determined using a Nanodrop 8000 (Thermo Fisher Scientific, Waltham, MA, USA) and gel electrophoresis.

### Quantitative PCR (qPCR)

Bacterial and fungal abundance were estimated with qPCR using a Bio-Rad CFX detection system (Bio-Rad Laboratories, Inc.). Bacterial 16S rRNA was targeted with the 338F-518R^69^ and fungal 18S rRNA with the primers FR1-FF390^70^. Each reaction (20 µL final volume) consisted of 400 nM reverse and forward primers, 50× diluted template (according to inhibition tests), molecular biology grade water, and 1× SsoFast™ EvaGreen^®^ Supermix for bacteria or 1× BioRad iQ™SYBR^®^ Supermix for fungi (Bio-Rad Laboratories, Inc.). Template concentration was optimized via inhibition tests using M13 template and primers. The thermal profile for bacteria was as follows: initial denaturation at 98 °C for 2 min followed by 40 cycles of 98 °C for 5 s and annealing at 55 °C for 5 s. The thermal profile for fungi comprised an initial denaturation at 95 °C for 5 min, 40 cycles of 95 °C for 15 s, annealing at 50 °C for 30 s, and extension at 70 °C for 45 s. For both amplicons, the analysis was followed by a melt curve analysis (41 cycles of 5 s at 65–95 °C) was carried out to verify specific target amplification. Standard curves for quantification were constructed using serial dilutions of plasmid DNA containing the cloned target gene (*Clostridium thermocellum* for bacterial 16S rRNA and environmental fungal genomic DNA). All qPCR assays included no template controls and were optimized to ensure reaction efficiencies of 95–110% and R^2^ values between 0.99 and 1.00.

### DNA sequencing and bioinformatics

High-throughput sequencing (2 × 250 bp) was performed by Génome Québec (McGill University, Montreal, QC) on an Illumina MiSeq platform. Amplicon libraries were prepared as recommended by the Earth Microbiome Project (https://earthmicrobiome.org). For prokaryotes, we used the primers 515F (5′-GTGCCAGCMGCCGCGGTAA-3′) and 806R (5′-GGACTACHVGGGTWTCTAAT-3′) targeting the V4 region of the 16S rRNA gene^71^. The fungal ITS region was amplified using ITS1f (5′-CTTGGTCATTTAGAGGAAGTAA-3′) paired with ITS2 (5′-GCTGCGTTCTTCATCGATGC-3′)^72^.

Bioinformatics were carried out in QIIME 2 2020.6^73^. Firstly, we used q2‐dada2 denoise-paired for denoising, dereplication, chimera filtering and merging of demultiplexed paired-end reads^74^. Because fungal ITS sequences present length polymorphism, they were previously trimmed using q2-itsxpress^75^, which finds and trims the ends of the ITS region. We obtained a total of 3,141,597 quality filtered and trimmed reads and 32,555 amplicon sequence variants (ASVs) for 16S rRNA, and 3,564,848 reads and 2079 ASVs for ITS. For 16S rRNA data, we used q2-phylogeny align-to-tree-mafft-fasttree to align ASVs and construct a phylogenetic tree ^76,77^. Phylogenetic analyses were not carried out on ITS sequences, as their higher variability makes them unreliable for this type of analysis. Taxonomy was assigned using classify‐sklearn naïve Bayes in q2‐feature‐classifier^78^ with taxonomic classifiers trained against the prokaryotic reference sequences from Silva v. 138 with 99% OTUs^79^ and the fungal reference sequences from UNITE v. 8.2 with dynamic use of clustering thresholds^80^. The database FungalTraits helped us inquire on the functional traits of sensitive fungal taxa^54^.

### Complementary data: plant growth and soil properties

Microbial data was related to both CC biomass and early crop biomass of the following year’s crop (tomato). Cc aboveground biomass was measured on 11 November 2015, before severe frost conditions^26^. Within each sub-plot, aboveground biomass from living and dead plants was collected from two randomly located quadrants (0.25 m^2^)^27^. No weed biomass was sampled due to negligible amounts. Early crop biomass was sampled in the following spring (17 June 2016) by harvesting aboveground biomass of 6 tomato plants from the inside guard row per sub-plot^6^. All plant biomass samples were dried at 60 °C to express data on a dry weight basis (kg ha^−1^). For further detail regarding plant growth measurements refer to previous publications^6,26,27^*.*

Soil physicochemical data was obtained from parallel studies carried out in the trial. Soil nitrate, ammonium, SOC, total N and C:N were measured in April 2016 (before tillage) and texture in September 2016 (at tomato harvest). All soil measurements were carried out on composite samples of 6 cores (0–15 cm) obtained at each sub-plot as described in Chahal and Van Eerd^27^. Results are available in previous publications^26,27^ and Table S8.

### Data analysis

Data analysis was carried out in QIIME 2 and R v. 3.6.3^81^, and graphics were made using GraphPad Prism9, ‘ggplot2’ in R and Inkscape v. 1.0.2-2. Alpha and beta diversity analyses (within- and between-sample, respectively) were carried out on rarefied ASV tables to avoid biases caused by differences in library size (Fig. S5). Microbial richness was analyzed both as ASVs and genera counts, and with a phylogenetic index (Faith PD), while ASV and genera evenness were measured using Pielou’s index. These indices, as well as microbial abundance data (log copies g^−1^ dry soil) from qPCR, were analyzed using ANOVA with linear mixed-effect models to account for the hierarchical nature of the experimental design as random effects (block > plot > split-plot). Tukey pairwise comparisons were carried out using package ‘multcomp’. Residuals were tested for normality and homogeneity of variance, and variance structures were applied if the latter was not satisfied.

Community composition and beta diversity were analyzed in the R package ‘vegan’^82^ using presence-absence metrics, which resulted more sensitive to these longer term treatments. We used the phylogenetic distance metric unweighted UniFrac for prokaryotes^47^ and Jaccard distance for fungi. Permutational multivariate ANOVA (PERMANOVA) was used to test the effect of CC and residue management on community composition, previously assessing beta dispersion with *betadisper*. A PERMANOVA with restricted permutations was carried out with *adonis2* following recommendations for split-plot designs by Anderson et al*.*^83^. Because there was a high proportion of unexplained variability, canonical analysis of principal coordinates (CAP) allowed us to uncover and visualize treatment-driven patterns that were masked by other sources of variability^84^. This analysis was run using the function *capscale*, removing block effects (partial CAP) to focus on treatment effects. For prokaryotes, residue management effects were also set as condition as they had a negligible influence.

Finally, taxonomic changes were explored using two methods: indicator species analysis in package ‘indicspecies’^85^ and ANOVA-like differential expression (ALDEx) in package ‘ALDEx2’^86^. All analyses were carried out previously filtering highly rare taxa (less than 10 reads in total and/or present in less than 2 samples). Indicator species analysis was carried out using *multipatt* (multi-level pattern analysis) to analyze taxa distribution patterns among treatments. Changes in the relative abundance of taxa between CCs were analyzed using *aldex.kw* and *aldex.corr* correlations with CAP axes, while *aldex.t* was used to test residue management effects. We also used *aldex.corr* to explore correlations between the relative abundance of taxa and early crop growth.

### Plant material

All plant material in the study was collected from long-term field experiments at the Ontario Crops Research Centre at Ridgetown Campus, University of Guelph. Plants were collected in accordance with University of Guelph and Ontario Ministry of Agriculture, Food and Rural Affairs guidelines.

## Supplementary Information

Supplementary Information.

## Data Availability

Raw sequencing data have been deposited in the Sequence Read Archive (SRA) of the National Centre for Biotechnology Information (NCBI) under BioProject ID PRJNA798719.
