# Author Correction: Cover crop-driven shifts in soil microbial communities could modulate early tomato biomass via plant-soil feedbacks

**DOI:** 10.1038/s41598-022-18010-4

**Published:** 2022-08-05

**Authors:** Micaela Tosi, John Drummelsmith, Dasiel Obregón, Inderjot Chahal, Laura L. Van Eerd, Kari E. Dunfield

**Affiliations:** 1 School of Environmental Sciences, University of Guelph, 50 Stone Rd. E, Guelph, ON N1G 2W1 Canada; 2 School of Environmental Sciences, University of Guelph, Ridgetown Campus, Ridgetown, ON N0P 2C0 Canada

Correction to: *Scientific Reports* 10.1038/s41598-022-11845-x, published online 01 June 2022

The original version of this Article contained an error in the legend of Figure 5.

“Figure 5. Soil prokaryotic (blue) and fungal (red) phyla correlated with early tomato growth in the following year. In each plot, Spearman correlation coefficients (r_s_) and P values from ALDEx (*aldex.corr*) are shown. If applicable, genera correlated to early crop growth are shown within each phylum’s plot (see Table S7 for full id.). Phyla names in bold were also sensitive to CCs (see Fig. 2c,f). Symbols between brackets represent Benjamini–Hochberg corrected P values, when significant: ***< 0.001, **< 0.01, *< 0.05, < 0.10.”

now reads:

“Figure 5. Soil prokaryotic and fungal phyla positively (blue) and negatively (red) correlated with early tomato growth in the following year. In each plot, Spearman correlation coefficients (r_s_) and P values from ALDEx (*aldex.corr*) are shown. If applicable, genera correlated to early crop growth are shown within each phylum’s plot (see Table S7 for full id.). Phyla names in bold were also sensitive to CCs (see Fig. 2c,f). Symbols between brackets represent Benjamini–Hochberg corrected P values, when significant: ***< 0.001, **< 0.01, *< 0.05, < 0.10.”

The original Article has been corrected.

