# Supplementary material for: Cover crop-driven shifts in soil microbial communities could modulate early tomato biomass via plant-soil feedbacks
Source: Sci Rep. 2022 Jun 1;12:9140. doi: 10.1038/s41598-022-11845-x (PMC9160062; doi:10.1038/s41598-022-11845-x)
Supplement: Supplementary file 1 — Supplementary Information. [file 41598_2022_11845_MOESM1_ESM.pdf]

SUPPLEMENTARY MATERIAL

Cover crop-driven shifts in soil microbial communities could modulate early tomato biomass via plant-soil feedbacks

Micaela Tosi[1], John Drummelsmith[1], Dasiel Obregon Alvarez[1], Inderjot Chahal[2], Laura L. Van Eerd[2], Kari E. Dunfield[1*]

[1] School of Environmental Sciences, University of Guelph, 50 Stone Rd. E, Guelph, ON N1G 2W1, Canada
[2] School of Environmental Sciences, University of Guelph, Ridgetown Campus, Ridgetown, ON N0P 2C0, Canada
** Corresponding author: dunfield@uoguelph.ca*

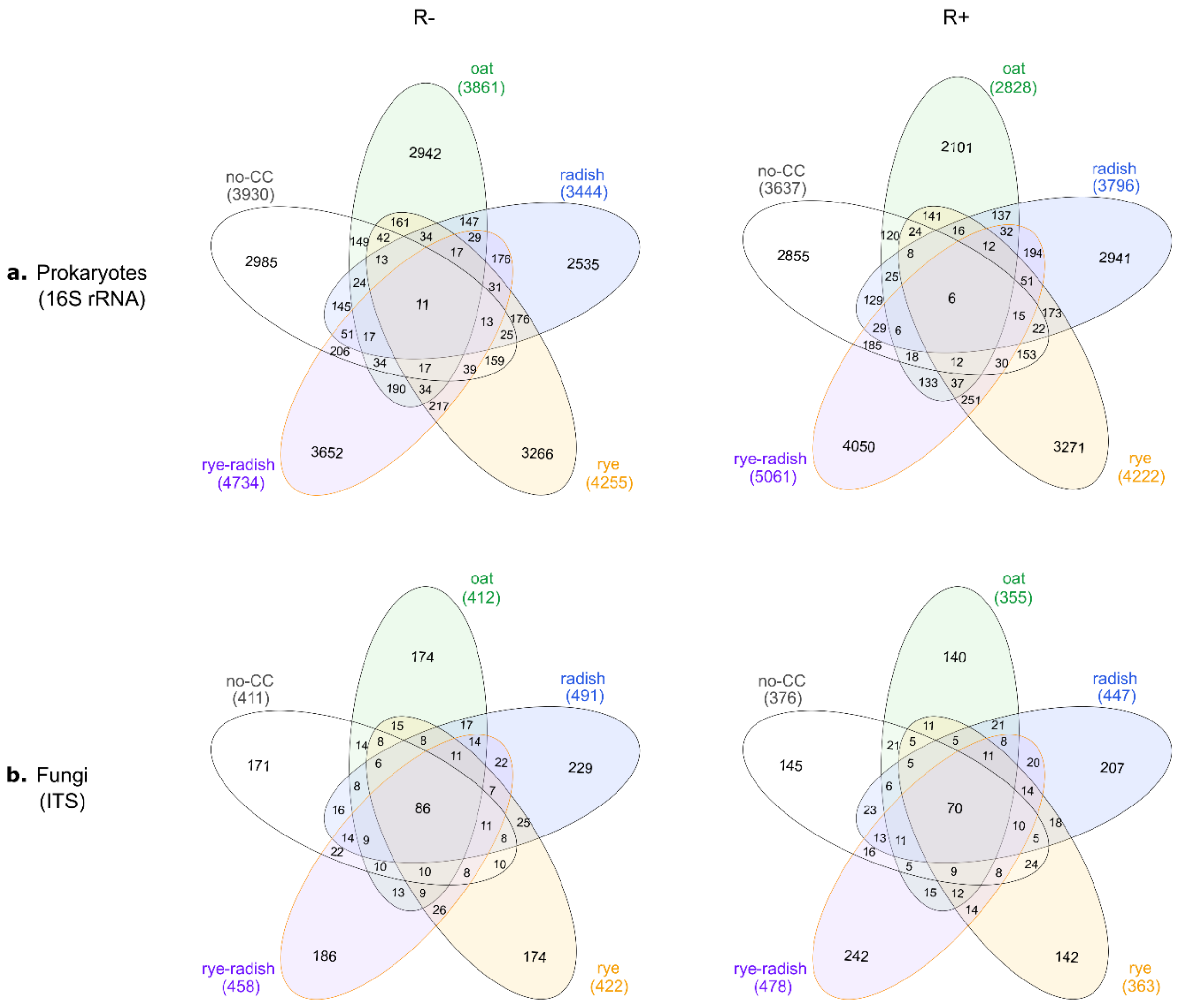

Fig. S1. Venn diagrams showing shared/unique prokaryotic (a) and fungal (b) ASVs between soils with different cover crops (CCs) and residue management. Calculations made from rarefied ASV table, for consistency with diversity analyses. Colors indicate different CC types and R-/R+ refers to main crop residues absent or present, respectively. Diagrams created with InteractiVenn (interactivenn.net) and figure was compiled and edited using Inkscape v. 1.0.2-2 (inkscape.org).

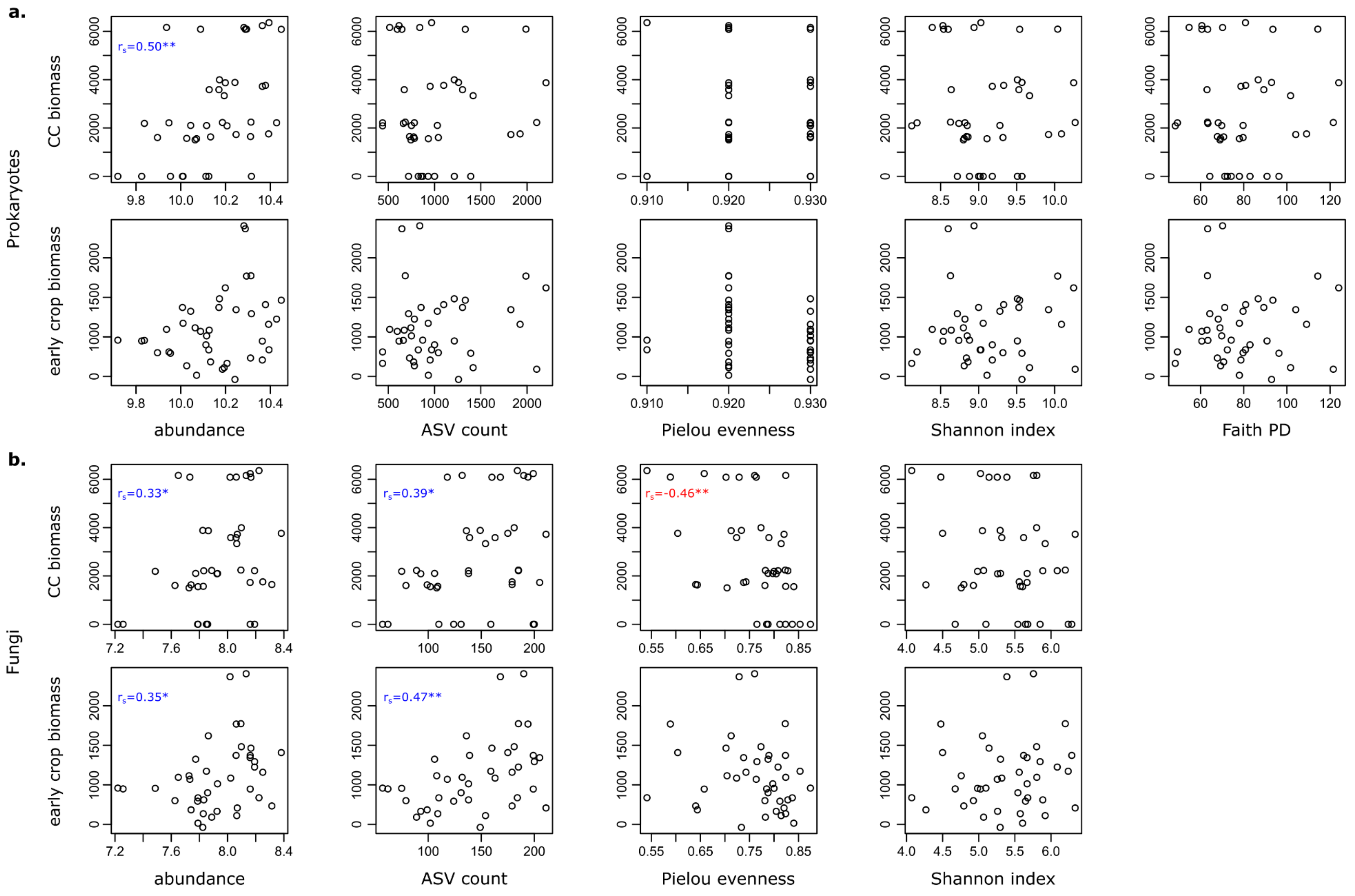

Fig. S2. Scatterplots showing relationships of soil microbial abundance and alpha diversity with cover crop (CC) and early crop biomass (fall 2015 and spring 2016, respectively). Data for prokaryotic (a) and fungal (b) communities are shown. Abundance refers to log gene copies per gram dry soil (bacterial 16S rRNA and fungal 18S rRNA). Spearman correlation coefficients (r_s) are shown only when significant (** P<0.01, * P<0.05).

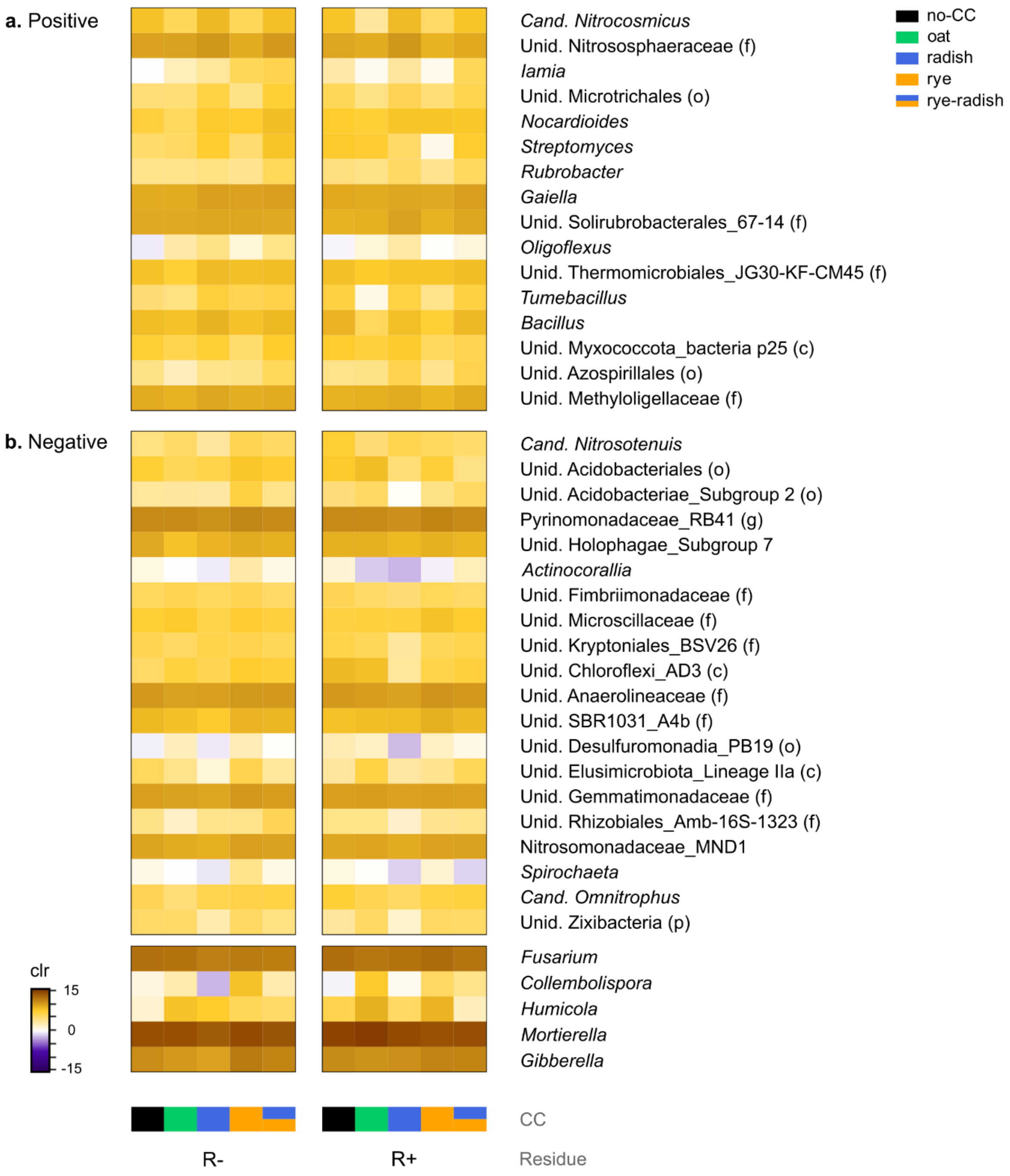

Fig. S3. Changes in the relative abundance (centered log-ratio or clr) of soil microbial taxa that were both sensitive to cover crop (CC) treatments and positively (a) or negatively (b) correlated with early crop growth. Mean values of four field replicates are shown. In taxa that could not be identified at the genus level, letters between brackets indicate the lowest taxonomic level available (p: phylum, c: class, o:order: f:family). See Table S7 for complete id. and correlation results. Colors at the bottom of the heatmap indicate different CCs and R-/R+ refers to main crop residues absent or present, respectively. Heatmaps created with R package 'gplots' (github.com/talgalili/gplots) and figure compiled and edited using Inkscape v. 1.0.2-2 (inkscape.org).

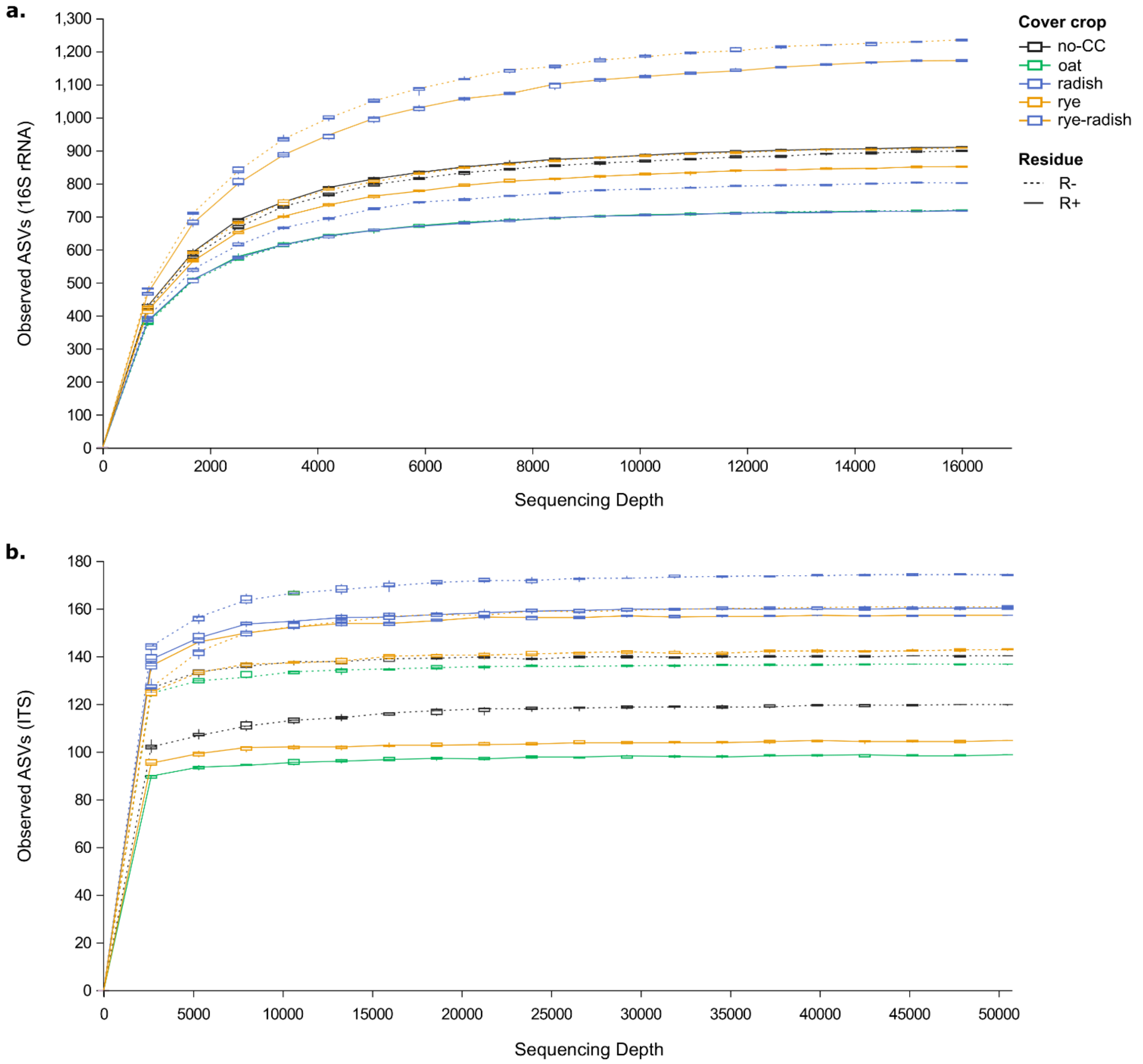

Fig. S4. Alpha rarefaction curves for prokaryotic 16S rRNA (a) and fungal ITS (b) sequencing data. Colors indicate different cover crops (CCs) and R-/R+ refers to main crop residues absent or present, respectively. Each curve connects the median of ASV counts across sampling depths, while the boxplots at each point represent the distribution of this variable within each group and at each sampling depth. Modified from q2-diversity alpha-rarefaction output using Inkscape v. 1.0.2-2 (inkscape.org). *Note: Rarefaction depth was 16,600 for 16S rRNA and 50,500 for ITS. Even though 16S rRNA rarefaction depth was comparatively low, curves show alpha diversity was fully surveyed.*

Table S1. Microbial alpha diversity indices in soils under contrasting cover crop (CC) and residue management (R-: absent, R+: present) treatments.

	R-															R+														
	**no-CC**			**oats**			**radish**			**rye**			**rye-radish**			**no-CC**			**oats**			**radish**			**rye**			**rye-radish**		
**Prokaryotes**																														
Faith PD	89.4	a	A	88.5	a	A	84.3	a	A	92.0	a	A	98.7	a	A	85.0	a	A	74.5	a	A	84.6	a	A	89.8	a	A	100.5	a	A
Richness	1017	a	A	1005	a	A	896	a	A	1103	a	A	1224	a	A	944	a	A	729	a	A	987	a	A	1098	a	A	1317	a	A
Shannon Index	9.13	a	A	9.00	a	A	8.95	a	A	9.23	a	A	9.50	a	A	9.12	a	A	8.74	a	A	8.99	a	A	9.22	a	A	9.43	a	A
**Fungi**																														
Richness	135	a	A	138	a	A	178	a	A	143	a	A	161	a	A	126	a	A	115	a	A	159	a	A	122	a	A	166	a	A
Shannon Index	5.82	a	A	5.71	a	A	4.91	a	A	5.24	a	A	5.30	a	A	5.44	a	A	5.41	a	A	5.32	a	A	5.05	a	A	5.66	a	A
**F:B ratio**	0.78	a	A	0.63	a	A	0.59	a	A	0.75	a	A	0.75	a	A	0.51	a	B	0.52	a	A	0.56	a	A	0.53	a	B	0.62	a	A

Abundance data was obtained from qPCR was expressed as copies of 16S rRNA or 18S rRNA gene (bacteria and fungi, respectively) per gram dry soil.

All diversity indices calculated from ASV table.

Different lowercase letters show significant differences between CCs within residue treatments and different uppercase letters show differences between residue management practices within CC treatments (Tukey HSD test, alpha=0.05).

Table S2. PERMANOVA results (*adonis2*) showing effect of cover crop (CC) and residue management treatments on soil prokaryotic and fungal community composition (unweighted UniFrac and Jaccard distance, respectively).

		**df**	**SS**	**R^2**	**F**	**Pr(>F)**	
Prokaryotes	block	3	0.37	0.21	1.41	0.003	**
(unweighted UniFrac)	CC	4	0.38	0.21	1.07	0.215	
	whole-plot error	12	1.05	0.58			
	whole-plot total	19	1.80	1.00			
	residue	1	0.15	0.02	0.94	0.643	
	CC:residue	4	0.61	0.09	0.96	0.699	
	split-plot error	15	2.41	0.35			
	total	39	6.78	1.00			
Fungi	block	3	0.66	0.18	1.20	0.003	**
(Jaccard)	CC	4	0.80	0.22	1.09	0.042	*
	whole-plot error	12	2.21	0.60			
	whole-plot total	19	3.68	1.00			
	residue	1	0.36	0.03	1.05	0.154	
	CC:residue	4	1.39	0.10	1.02	0.269	
	split-plot error	15	5.14	0.36			
	total	39	14.24	1.00			

Signif. codes: 0 '***' 0.001 '**' 0.01 '*' 0.05 '.' 0.1 ' ' 1

Restricted permutations were carried out following recommendations by Anderson et al. (2008) for split-plot designs. For that purpose, CC effects were tested at the whole-plot level, while residue and the CC:residue interaction were tested at the split-plot level. Random block effects were also considered.

Quantitative metrics showed similar results for prokaryotes (unweighted UniFrac). For fungi, Bray-Curtis detected a significant effect of CC (R^2=0.23, P=0.024) but a marginal effect of residue management (R^2=0.03, P=0.071).

Table S3. Indicator soil prokaryotic taxa for different cover crops (CCs) in the whole data set (both), residue absent only (R-) and residue present only (R+).

**Dataset**	**Taxon**	**no-CC**	**oat**	**radish**	**rye**	**rye-radish**	**P-value**
Both	p_Verrucomicrobiota;c_Verrucomicrobiae;o_Opitutales;f_Puniceicoccaceae;g_*Cerasicoccus*	x					*
	p_Myxococcota;c_Polyangia;o_UASB-TL25		x				**
	p_Verrucomicrobiota;c_Verrucomicrobiae;o_Pedosphaerales;f_Pedosphaeraceae;g_Ellin517 [3]			x			*
	p_Firmicutes;c_Clostridia;o_Eubacteriales;f_Alkalibacteraceae;g_*Alkalibacter*				x		*
	p_Proteobacteria;c_Gammaproteobacteria;o_Coxiellales;f_Coxiellaceae;g_*Coxiella*					x	*
	p_Proteobacteria;c_Gammaproteobacteria;o_Diplorickettsiales;f_Diplorickettsiaceae;g_*Aquicella* [3]	x		x		x	*
	p_Gemmatimonadota;c_Longimicrobia;o_Longimicrobiales;f_Longimicrobiaceae;g_*Longimicrobiaceae* [2]	x		x		x	**
	p_Myxococcota;c_Polyangia;o_Polyangiales;f_Polyangiaceae	x		x		x	*
	d_Archaea; p_Thermoplasmatota;c_Thermoplasmata;o_Methanomassiliicoccales [1]	x			x	x	**
	p_Acidobacteriota;c_Vicinamibacteria;o_Vicinamibacterales;f_Vicinamibacteraceae;g_*Luteitalea*	x			x	x	*
	p_Planctomycetota;c_Phycisphaerae;o_mle1-8		x		x	x	*
	p_Actinobacteriota;c_Actinobacteria;o_Micrococcales;f_Intrasporangiaceae;g_*Terrabacter*		x		x	x	**
	p_Zixibacteria [3]	x	x		x	x	**
	p_Desulfobacterota;c_Desulfuromonadia;o_PB19 [2]	x	x		x	x	*
	p_Actinobacteriota;c_Acidimicrobiia;o_Microtrichales;f_Ilumatobacteraceae [3]	x		x	x	x	*
	p_Firmicutes;c_Bacilli;o_Alicyclobacillales;f_Alicyclobacillaceae;g_*Tumebacillus* [2]	x		x	x	x	*
	p_Bacteroidota;c_Bacteroidia;o_Chitinophagales;f_Saprospiraceae [2]	x		x	x	x	*
	p_Planctomycetota;c_Planctomycetes;o_Pirellulales;f_Pirellulaceae;g_*Blastopirellula* [2]	x		x	x	x	*
	p_Proteobacteria;c_Alphaproteobacteria;o_Rhizobiales;f_Labraceae;g_*Labrys* [3]	x		x	x	x	*
	p_Bacteroidota;c_Bacteroidia;o_Flavobacteriales;f_NS9_marine_group		x	x	x	x	*
R-	p_Abditibacteriota;c_Abditibacteria;o_Abditibacteriales;f_Abditibacteriaceae;g_*Abditibacterium*	x					*
	p_Patescibacteria;c_Gracilibacteria		x				*
	p_Fibrobacterota;c_Fibrobacteria;o_Fibrobacterales;f_Fibrobacteraceae		x				*
	p_Bacteroidota;c_Bacteroidia;o_Chitinophagales;f_Chitinophagaceae;g_*Taibaiella*		x				*
	p_Actinobacteriota;c_Actinobacteria;o_Propionibacteriales;f_Nocardioidaceae;g_*Aeromicrobium*				x		*
	p_Actinobacteriota;c_Actinobacteria;o_Micromonosporales;f_Micromonosporaceae;g_*Actinoplanes* [1]					x	**
	p_Proteobacteria;c_Alphaproteobacteria;o_Rhizobiales;f_Methyloligellaceae	x			x		*
	p_Myxococcota;c_Polyangia;o_Polyangiales;f_Sandaracinaceae	x	x		x	x	*
	p_Firmicutes;c_Clostridia;o_Clostridiales;f_Clostridiaceae;g_*Clostridium_sensu_stricto_13*	x		x	x	x	*
R+	p_Proteobacteria;c_Gammaproteobacteria;o_Burkholderiales			x			*
	p_Actinobacteriota;c_Acidimicrobiia;o_Microtrichales;f_Ilumatobacteraceae;g_CL500-29_marine_group	x		x			*
	p_Dependentiae;c_Babeliae;o_Babeliales;f_Babeliaceae;g_*Babeliaceae*	x				x	*
	p_Firmicutes;c_Bacilli;o_Paenibacillales;f_Paenibacillaceae;g_*Ammoniphilus*			x		x	*
	p_Chloroflexi;c_Chloroflexia;o_Chloroflexales;f_Chloroflexaceae;g_FFCH7168				x	x	*
	p_Proteobacteria;c_Alphaproteobacteria;o_Rhizobiales;f_A0839	x			x	x	*
	p_Patescibacteria;c_Gracilibacteria;o_Candidatus_Peregrinibacteria	x			x	x	**
	p_Chloroflexi;c_AD3	x	x		x	x	*
	p_Proteobacteria;c_Alphaproteobacteria;o_Dongiales;f_Dongiaceae;g_*Dongia*	x		x	x	x	*
	d_Archaea;p_Thermoplasmatota;c_Thermoplasmata	x		x	x	x	*

Results from multilevel pattern analysis (package “indispec”), association function (IndVal.g, alpha=0.05). The “x” indicates CC treatments where each taxon was considered an indicator.

Taxonomic levels shown as: d_: domain, p_: phylum, c_: class, o_: order, f_: family, g_: genus. Domain only show for Archaea (otherwise it belongs to Bacteria).

P-values are represented with asterisks: <0.01 (**), <0.05 (*).

Numbers next to taxa indicate they were also detected by other analyses: [1] *aldex.kw*, [2] *aldex.corr* with CAP axes, [3] both.

Table S4. Soil prokaryotic taxa correlated with first two CAP axes (Fig. 2a) according to *aldex.corr* or sensitive to cover crops (CCs) according to *aldex.kw*. CAP1: rye-radish to oat, CAP2: rye to radish.

			**Pearson**		**Spearman**	
**Taxon**	**Axes**	**Corr**	**r**	**P**	**r_s**	**P**
d_Archaea,p_Aenigmarchaeota,c_Aenigmarchaeia,o_Aenigmarchaeales,f_Aenigmarchaeales,g_Aenigmarchaeales	2	-	-0.25	0.166	-0.33	0.047
d_Archaea,p_Crenarchaeota,c_Nitrososphaeria,o_Nitrosopumilales,f_Nitrosopumilaceae,g_Cand_Nitrosotenuis	2	-	-0.32	0.053	-0.39	0.016
d_Archaea,p_Crenarchaeota,c_Nitrososphaeria,o_Nitrososphaerales,f_Nitrososphaeraceae,g_Cand_Nitrocosmicus	1,2	-	-0.39	0.020	0.03	0.816
d_Archaea,p_Crenarchaeota,c_Nitrososphaeria,o_Nitrososphaerales,f_Nitrososphaeraceae,g_Cand_Nitrososphaera	1	-	-0.38	0.030	-0.30	0.069
**d_Archaea,p_Crenarchaeota,c_Nitrososphaeria,o_Nitrososphaerales,f_Nitrososphaeraceae (…)**	**2**	**+**	**0.53**	**0.000**	**0.49**	**0.002**
d_Archaea,p_Nanoarchaeota,c_Nanoarchaeia,o_Woesearchaeales (…)	1	-	-0.42	0.012	-0.41	0.011
d_Archaea,p_Thermoplasmatota,c_Thermoplasmata,_,_,_	1	-	-0.43	0.009	-0.18	0.268
d_Archaea,p_Thermoplasmatota,c_Thermoplasmata,o_Marine_Group_II (…) (*aldex.kw*)						
d_Archaea,p_Thermoplasmatota,c_Thermoplasmata,o_Methanomassiliicoccales,f_unc (…) (*aldex.kw*) [2]						
p_Acidobacteriota,c_Acidobacteriae,o_Acidobacteriales,f_unc,g_unc	2	-	-0.43	0.006	-0.44	0.006
**p_Acidobacteriota,c_Acidobacteriae,o_Subgroup_2 (…)**	**2**	**-**	**-0.44**	**0.008**	**-0.55**	**0.000**
p_Acidobacteriota,c_Blastocatellia,o_11-24 (…)	2	-	-0.25	0.127	-0.39	0.018
p_Acidobacteriota,c_Blastocatellia,o_Blastocatellales,f_Blastocatellaceae,g_JGI_0001001-H03	2	-	-0.28	0.101	-0.38	0.017
p_Acidobacteriota,c_Blastocatellia,o_Pyrinomonadales,f_Pyrinomonadaceae,g_RB41	2	-	-0.49	0.002	-0.46	0.003
p_Acidobacteriota,c_Holophagae,o_Subgroup_7 (…)	2	-	-0.38	0.015	-0.39	0.014
p_Actinobacteriota,c_Acidimicrobiia,_,_,_	2	+	0.40	0.017	0.31	0.057
p_Actinobacteriota,c_Acidimicrobiia,o_Actinomarinales,f_unc,g_unc	1,2	-	-0.37	0.031	-0.42	0.009
p_Actinobacteriota,c_Acidimicrobiia,o_IMCC26256 (…)	1	-	-0.38	0.016	-0.12	0.466
p_Actinobacteriota,c_Acidimicrobiia,o_Microtrichales,f_Iamiaceae,g_Iamia	1	-	-0.39	0.023	-0.34	0.035
p_Actinobacteriota,c_Acidimicrobiia,o_Microtrichales,f_Ilumatobacteraceae,g_unc [3]	1	-	-0.37	0.031	-0.17	0.292
p_Actinobacteriota,c_Acidimicrobiia,o_Microtrichales,f_unc (…)	2	+	0.42	0.009	0.27	0.093
p_Actinobacteriota,c_Actinobacteria,o_Corynebacteriales,f_Mycobacteriaceae,g_Mycobacterium	2	+	0.33	0.040	0.25	0.132
p_Actinobacteriota,c_Actinobacteria,o_Micrococcales,f_Microbacteriaceae,g_Cryobacterium	2	+	0.50	0.003	0.50	0.002
p_Actinobacteriota,c_Actinobacteria,o_Micromonosporales,f_Micromonosporaceae,g_Actinoplanes (*aldex.kw*) [2]						
p_Actinobacteriota,c_Actinobacteria,o_Micromonosporales,f_Micromonosporaceae,g_Dactylosporangium	1	-	-0.41	0.017	-0.37	0.021
p_Actinobacteriota,c_Actinobacteria,o_Propionibacteriales,f_Nocardioidaceae,g_Nocardioides	1,2	-	-0.39	0.014	-0.13	0.451
p_Actinobacteriota,c_Actinobacteria,o_Streptomycetales,f_Streptomycetaceae,g_Streptomyces	2	+	0.38	0.025	0.40	0.012
p_Actinobacteriota,c_Actinobacteria,o_Streptosporangiales,f_Streptosporangiaceae,g_Nonomuraea	2	+	0.43	0.015	0.47	0.004
p_Actinobacteriota,c_Actinobacteria,o_Streptosporangiales,f_Streptosporangiaceae,g_Streptosporangium	1	-	-0.43	0.017	-0.51	0.003
p_Actinobacteriota,c_Actinobacteria,o_Streptosporangiales,f_Thermomonosporaceae,g_Actinocorallia	1	-	-0.35	0.060	-0.39	0.022
p_Actinobacteriota,c_Rubrobacteria,o_Rubrobacterales,f_Rubrobacteriaceae,g_Rubrobacter	2	+	0.33	0.054	0.33	0.046
p_Actinobacteriota,c_Thermoleophilia,o_Gaiellales,f_Gaiellaceae,g_Gaiella	2	+	0.45	0.004	0.42	0.008
p_Actinobacteriota,c_Thermoleophilia,o_Solirubrobacterales,f_67-14 (…)	2	+	0.46	0.003	0.40	0.011
p_Actinobacteriota,c_Thermoleophilia,o_Solirubrobacterales,f_Solirubrobacteraceae,g_Solirubrobacter	1	-	-0.35	0.030	-0.22	0.186
p_Armatimonadota,c_Chthonomonadetes,o_Chthonomonadales,f_Chthonomonadales,g_Chthonomonadales	2	-	-0.26	0.118	-0.35	0.033
p_Armatimonadota,c_Fimbriimonadia,o_Fimbriimonadales,f_Fimbriimonadaceae,g_Fimbriimonadaceae	2	-	-0.41	0.010	-0.42	0.010
p_Armatimonadota,c_unc (…)	2	-	-0.32	0.048	-0.30	0.078
p_Bacteroidota,c_Bacteroidia,o_Chitinophagales,f_Chitinophagaceae,g_Terrimonas	2	-	-0.30	0.064	-0.36	0.027
p_Bacteroidota,c_Bacteroidia,o_Chitinophagales,f_Saprospiraceae,g_unc [2]	1	-	-0.38	0.023	-0.26	0.115
p_Bacteroidota,c_Bacteroidia,o_Cytophagales,f_Microscillaceae,g_unc	2	-	-0.45	0.004	-0.38	0.019
p_Bacteroidota,c_Bacteroidia,o_Sphingobacteriales,f_env.OPS_17 (…)	2	-	-0.25	0.146	-0.43	0.009
p_Bacteroidota,c_Kapabacteria,o_Kapabacteriales,f_Kapabacteriales,g_Kapabacteriales	1	+	0.33	0.053	0.42	0.011
p_Bacteroidota,c_Kryptonia,o_Kryptoniales,f_BSV26 (…)	2	-	-0.36	0.025	-0.32	0.056

p_Bdellovibrionota,c_Oligoflexia,o_Oligoflexales,f_Oligoflexales,g_Oligoflexus	2	+	0.41	0.023	0.42	0.012
p_Chloroflexi,c_AD3 (…)	2	-	-0.32	0.051	-0.39	0.013
p_Chloroflexi,c_Anaerolineae,o_Anaerolineales,f_Anaerolineaceae,g_unc	2	-	-0.34	0.036	-0.35	0.029
p_Chloroflexi,c_Anaerolineae,o_Ardenticatenales,f_unc (…)	1	-	-0.44	0.005	-0.38	0.021
p_Chloroflexi,c_Anaerolineae,o_Caldilineales,f_Caldilineaceae,g_Litorilinea	1	-	-0.38	0.041	-0.44	0.007
p_Chloroflexi,c_Anaerolineae,o_RBG-13-54-9 (…)	2	-	-0.25	0.135	-0.34	0.038
p_Chloroflexi,c_Anaerolineae,o_SBR1031,f_A4b,g_A4b	1,2	-	-0.35	0.031	-0.30	0.066
p_Chloroflexi,c_Chloroflexia,o_Thermomicrobiales,f_JG30-KF-CM45 (…)	2	+	0.36	0.023	0.35	0.033
p_Chloroflexi,c_JG30-KF-CM66 (…) [1]	1	-	-0.48	0.002	-0.42	0.008
p_Chloroflexi,c_Ktedonobacteria,o_Ktedonobacterales,f_JG30-KF-AS9 (…)	2	-	-0.34	0.064	-0.43	0.010
p_Desulfobacterota,c_Desulfuromonadia,o_PB19 (…) [2]	2	-	-0.36	0.048	-0.36	0.034
p_Desulfobacterota,c_unc,o_unc,f_unc,g_unc	2	-	-0.27	0.120	-0.36	0.024
p_Elusimicrobiota,c_Elusimicrobia,o_Lineage_IV (…) (*aldex.kw*)						
p_Elusimicrobiota,c_Lineage_IIa,o_Lineage_IIa,f_Lineage_IIa,g_Lineage_IIa	2	-	-0.35	0.044	-0.36	0.023
p_Firmicutes,c_Bacilli,o_Alicyclobacillales,f_Alicyclobacillaceae,g_Tumebacillus [2]	1,2	-	-0.46	0.005	-0.19	0.246
p_Firmicutes,c_Bacilli,o_Bacillales,f_Bacillaceae,g_Bacillus	2	+	0.34	0.036	0.40	0.012
p_Firmicutes,c_Bacilli,o_Bacillales,f_Planococcaceae,g_Sporosarcina	2	+	0.36	0.045	0.38	0.029
p_Firmicutes,c_Bacilli,o_Paenibacillales,f_Paenibacillaceae,g_Paenibacillus	1,2	-	-0.35	0.046	-0.32	0.049
p_Firmicutes,c_Clostridia,o_Clostridiales,f_Clostridiaceae,g_Clostridium_sensu_stricto_13	2	+	0.19	0.268	0.34	0.037
p_GAL15,c_GAL15,o_GAL15,f_GAL15,g_GAL15	2	-	-0.35	0.054	-0.39	0.020
p_Gemmatimonadota,c_AKAU4049,o_AKAU4049,f_AKAU4049,g_AKAU4049	1,2	-	-0.32	0.066	-0.37	0.026
p_Gemmatimonadota,c_Gemmatimonadetes,o_Gemmatimonadales,f_Gemmatimonadaceae,g_unc	2	-	-0.40	0.012	-0.38	0.019
p_Gemmatimonadota,c_Longimicrobia,o_Longimicrobiales,f_Longimicrobiaceae (…) [2]	2	+	0.29	0.117	0.32	0.057
p_Gemmatimonadota,c_S0134_terrestrial_group (…)	1	-	-0.39	0.028	-0.41	0.011
**p_Latescibacterota,c_Latescibacterota (…)**	**2**	**-**	**-0.53**	**0.001**	**-0.50**	**0.001**
p_Methylomirabilota,c_Methylomirabilia,o_Rokubacteriales (…)	2	-	-0.34	0.037	-0.33	0.045
p_Myxococcota,c_bacteriap25,o_bacteriap25,f_bacteriap25,g_bacteriap25	2	+	0.33	0.043	0.16	0.320
p_Myxococcota,c_Myxococcia,o_Myxococcales,f_27F-1492R (…) (*aldex.kw*)						
p_Patescibacteria,c_Gracilibacteria,o_Cand_Peregrinibacteria (…)	1	-	-0.29	0.118	-0.38	0.022
p_Planctomycetota,c_Phycisphaerae,o_Phycisphaerales,f_Phycisphaeraceae,g_SM1A02	2	-	-0.31	0.065	-0.36	0.029
p_Planctomycetota,c_Phycisphaerae,o_Tepidisphaerales,f_WD2101_soil_group (…)	2	-	-0.46	0.003	-0.48	0.003
p_Planctomycetota,c_Pla3_lineage (…)	2	-	-0.29	0.079	-0.34	0.038
p_Planctomycetota,c_Pla4_lineage (…)	2	-	-0.32	0.047	-0.27	0.101
p_Planctomycetota,c_Planctomycetes,o_Gemmatales,f_Gemmataceae,g_Gemmata	2	+	0.39	0.014	0.35	0.032
p_Planctomycetota,c_Planctomycetes,o_Gemmatales,f_Gemmataceae,g_unc (*aldex.kw*)						
**p_Planctomycetota,c_Planctomycetes,o_Pirellulales,f_Pirellulaceae,g_Blastopirellula [2]**	**1**	**-**	**-0.51**	**0.003**	**-0.54**	**0.001**
p_Planctomycetota,c_Planctomycetes,o_Pirellulales,f_Pirellulaceae,g_Pir4_lineage	1,2	-	-0.45	0.009	-0.41	0.011
p_Planctomycetota,c_Planctomycetes,o_Planctomycetales,f_Rubinisphaeraceae,g_SH-PL14	2	+	0.50	0.002	0.47	0.003
p_Planctomycetota,c_Planctomycetes,o_unc (…) (*aldex.kw*)						
p_Proteobacteria,c_Alphaproteobacteria,_,_,_	2	+	0.35	0.029	0.33	0.051
p_Proteobacteria,c_Alphaproteobacteria,o_Azospirillales,f_unc (…)	2	+	0.34	0.040	0.36	0.028
p_Proteobacteria,c_Alphaproteobacteria,o_Micropepsales,f_Micropepsaceae,g_unc	2	-	-0.32	0.055	-0.46	0.003
p_Proteobacteria,c_Alphaproteobacteria,o_Rhizobiales,f_A0839,g_A0839	1	-	-0.36	0.041	-0.37	0.021
p_Proteobacteria,c_Alphaproteobacteria,o_Rhizobiales,f_Amb-16S-1323,g_Amb-16S-1323	1	-	-0.34	0.042	-0.25	0.122
p_Proteobacteria,c_Alphaproteobacteria,o_Rhizobiales,f_Hyphomicrobiaceae,g_Hyphomicrobium	1	-	-0.35	0.040	-0.14	0.394
p_Proteobacteria,c_Alphaproteobacteria,o_Rhizobiales,f_Hyphomicrobiaceae,g_Pedomicrobium	2	+	0.34	0.034	0.27	0.097
p_Proteobacteria,c_Alphaproteobacteria,o_Rhizobiales,f_Labraceae,g_Labrys [3]	1	-	-0.40	0.026	-0.37	0.025

p_Proteobacteria,c_Alphaproteobacteria,o_Rhizobiales,f_Methyloligellaceae,g_unc	2	+	0.50	0.001	0.48	0.002
p_Proteobacteria,c_Alphaproteobacteria,o_Rhizobiales,f_Rhizobiales_Incertae_Sedis,g_Nordella	1	-	-0.36	0.027	-0.14	0.404
**p_Proteobacteria,c_Alphaproteobacteria,o_Rhizobiales,f_Rhizobiales_Incertae_Sedis,g_unc**	**1**	**-**	**-0.49**	**0.003**	**-0.59**	**0.000**
p_Proteobacteria,c_Alphaproteobacteria,o_Sphingomonadales,f_Sphingomonadaceae,g_Sphingomonas	1	-	-0.40	0.015	0.04	0.805
p_Proteobacteria,c_Alphaproteobacteria,o_unc,f_unc,g_unc	2	-	-0.47	0.003	-0.41	0.011
p_Proteobacteria,c_Gammaproteobacteria,_,_,_ (*aldex.kw*)						
**p_Proteobacteria,c_Gammaproteobacteria,o_Burkholderiales,f_A21b,g_A21b**	2	-	**-0.44**	**0.008**	**-0.54**	**0.000**
p_Proteobacteria,c_Gammaproteobacteria,o_Burkholderiales,f_Comamonadaceae,g_Rhizobacter	1	+	0.17	0.327	0.33	0.040
p_Proteobacteria,c_Gammaproteobacteria,o_Burkholderiales,f_Nitrosomonadaceae,g_Ellin6067	1	-	-0.37	0.027	-0.29	0.071
p_Proteobacteria,c_Gammaproteobacteria,o_Burkholderiales,f_Nitrosomonadaceae,g_IS-44	1	-	-0.39	0.015	-0.18	0.282
p_Proteobacteria,c_Gammaproteobacteria,o_Burkholderiales,f_Nitrosomonadaceae,g_mle1-7 [1]	1	-	-0.45	0.005	-0.05	0.744
p_Proteobacteria,c_Gammaproteobacteria,o_Burkholderiales,f_Nitrosomonadaceae,g_MND1	2	-	-0.48	0.002	-0.51	0.001
p_Proteobacteria,c_Gammaproteobacteria,o_Burkholderiales,f_Oxalobacteraceae,g_Massilia	2	-	-0.39	0.028	-0.40	0.014
p_Proteobacteria,c_Gammaproteobacteria,o_Burkholderiales,f_TRA3-20 (…)	1	-	-0.39	0.015	-0.26	0.122
p_Proteobacteria,c_Gammaproteobacteria,o_Diplorickettsiales,f_Diplorickettsiaceae,g_Aquicella [3]	1,2	-	-0.35	0.045	-0.30	0.065
p_Proteobacteria,c_Gammaproteobacteria,o_JG36-GS-52 (…)	1	-	-0.27	0.144	-0.33	0.055
p_RCP2-54 (…) [1]	2	-	-0.23	0.172	-0.38	0.018
p_Spirochaetota,c_Spirochaetia,o_Spirochaetales,f_Spirochaetaceae,g_Spirochaeta	2	-	-0.45	0.013	-0.52	0.002
p_Verrucomicrobiota,c_Chlamydiae,o_Chlamydiales,f_Parachlamydiaceae,g_Neochlamydia	2	+	0.38	0.027	0.40	0.015
p_Verrucomicrobiota,c_Omnitrophia,o_Omnitrophales,f_Omnitrophaceae,g_Cand_Omnitrophus	1,2	-	-0.39	0.014	-0.16	0.332
p_Verrucomicrobiota,c_Omnitrophia,o_Omnitrophales,f_Omnitrophales,g_Omnitrophales	1	-	-0.06	0.664	0.32	0.051
p_Verrucomicrobiota,c_Verrucomicrobiae,o_Chthoniobacterales,f_Chthoniobacteraceae,g_Cand_Udaeobacter	1	+	0.48	0.002	0.50	0.001
p_Verrucomicrobiota,c_Verrucomicrobiae,o_Chthoniobacterales,f_Xiphinematobacteraceae,g_Cand_Xiphinematobacter	2	+	0.31	0.054	0.39	0.014
p_Verrucomicrobiota,c_Verrucomicrobiae,o_Pedosphaerales,f_Pedosphaeraceae,g_Ellin517 [3]	2	+	0.35	0.046	0.33	0.064
p_Verrucomicrobiota,c_Verrucomicrobiae,o_Pedosphaerales,f_Pedosphaeraceae,g_Pedosphaeraceae	1,2	-	-0.33	0.039	-0.17	0.314
p_Verrucomicrobiota,c_Verrucomicrobiae,o_Pedosphaerales,f_Pedosphaeraceae,g_unc	2	-	-0.34	0.034	-0.34	0.036
p_Verrucomicrobiota,c_Verrucomicrobiae,o_S-BQ2-57_soil_group (…)	2	-	-0.28	0.099	-0.36	0.026
p_Zixibacteria (…) [3]	2	-	-0.49	0.003	-0.49	0.002

Taxonomic levels shown as: d_: domain, p_: phylum, c_: class, o_: order, f_: family, g_: genus. Domain only show for Archaea (otherwise it belongs to Bacteria).

Bold: BH-corrected P <0.10. Otherwise, P<0.05.

Numbers next to taxa indicate they were also detected by other analyses: [1] *aldex.kw*, [2] indicator species, [3] both.

(…): name was repeated until genus level, unc: uncultured.

Table S5. Indicator soil fungal taxa for different cover crops (CCs) in the whole data set (both), residue absent only (R-) and residue present only (R+).

**Dataset**	**Taxon**	**no-CC**	**oat**	**radish**	**rye**	**rye-radish**	**P-value**
Both	p_Olpidiomycota;c_Olpidiomycetes;o_Olpidiales;f_Olpidiaceae [2]			x			**
	p_Basidiomycota;c_Agaricomycetes;o_Agaricales;f_Entolomataceae;g_*Clitopilus*			x			**
	p_Ascomycota;c_Sordariomycetes;o_Glomerellales;f_Plectosphaerellaceae;g_*Acrostalagmus*					x	*
	p_Ascomycota;c_Dothideomycetes;o_Pleosporales;f_Leptosphaeriaceae;g_*Leptosphaeria*			x		x	*
	p_Ascomycota;c_Dothideomycetes;o_Pleosporales;f_Pleosporaceae;g_*Bipolaris* [2]				x	x	**
	p_Ascomycota;c_Dothideomycetes;o_Pleosporales;f_Didymosphaeriaceae;g_*Paraphaeosphaeria* [2]	x	x	x			*
	p_Ascomycota;c_Sordariomycetes;o_Hypocreales;f_Hypocreales_fam_Incertae_sedis;g_*Myxocephala* [1]	x			x	x	*
	p_Ascomycota;c_Leotiomycetes;o_Helotiales;f_Helotiales_fam_Incertae_sedis;g_*Collembolispora* [2]		x		x	x	**
	p_Chytridiomycota	x	x	x		x	*
	p_Ascomycota;c_Sordariomycetes;o_Hypocreales;f_Nectriaceae;g_*Neonectria* [2]		x	x	x	x	*
R-	p_Mortierellomycota;c_Mortierellomycetes;o_Mortierellales	x					*
	p_Ascomycota;c_Sordariomycetes;o_Hypocreales;f_Nectriaceae	x		x			**
	p_Basidiomycota;c_Agaricomycetes;o_Agaricales;f_Psathyrellaceae;g_*Coprinopsis*			x	x		*
	p_Ascomycota;c_Dothideomycetes;o_Venturiales;f_Sympoventuriaceae;g_*Ochroconis*	x		x	x		*
R+	p_Kickxellomycota;c_Kickxellomycetes;o_Kickxellales;f_Kickxellaceae;g_*Ramicandelaber*	x					*
	p_Rozellomycota;c_Rozellomycotina_cls_Incertae_sedis;o_GS11	x					*
	p_Ascomycota;c_Pezizomycetes;o_Pezizales;f_Ascodesmidaceae;g_*Cephaliophora*				x		*
	p_Glomeromycota					x	**
	p_Basidiomycota;c_Agaricomycetes		x	x		x	*
	p_Ascomycota;c_Leotiomycetes;o_Helotiales;f_Helotiaceae;g_*Tetracladium*	x		x	x	x	*

Results from multilevel pattern analysis (package "indispec"), association function (IndVal.g, alpha=0.05). The "x" indicates CC treatments where each taxon was considered an indicator.
Taxonomic levels shown as: p_: phylum, c_: class, o_: order, f_: family, g_: genus.
P-values are represented with asterisks: <0.01 (**), <0.05 (*).
Numbers next to taxa indicate they were also detected by other analyses: [1] *aldex.corr* with CAP axes, [2] both.

Table S6. Soil fungal taxa correlated with first two CAP axes (Fig. 2d). CAP1: oat to radish-based, CAP2: rye-based to other.

	Lifestyle				Pearson		Spearman	
**Taxon**	**Primary**	**Secondary**	**Axes**	**Corr**	**r**	**P**	**r_s**	**P**
p_Ascomycota,c_Dothideomycetes,o_Pleosporales,f_Didymosphaeriaceae,g_*Paraphaeosphaeria* [3]	wood_saprotroph		2	+	0.48	0.004	0.45	0.007
p_Ascomycota,c_Dothideomycetes,o_Pleosporales,f_Periconiaceae,g_*Periconia*	plant_pathogen	foliar_endophyte	1	-	-0.30	0.069	-0.41	0.010
**p_Ascomycota,c_Dothideomycetes,o_Pleosporales,f_Pleosporaceae,g_*Bipolaris*** [3]	plant_pathogen	litter_saprotroph	2	-	**-0.54**	**0.001**	**-0.55**	**0.001**
p_Ascomycota,c_Eurotiomycetes,o_Chaetothyriales,f_Herpotrichiellaceae,g_*Exophiala*	animal_parasite	litter_saprotroph	1	-	-0.14	0.416	-0.37	0.021
p_Ascomycota,c_Leotiomycetes,o_Helotiales,f_Helotiaceae,g_*Tetracladium*	litter_saprotroph		1	+	0.40	0.012	0.25	0.119
p_Ascomycota,c_Leotiomycetes,o_Helotiales,f_Helotiales_fam_Incertae_sedis,g_*Collembolispora* [3]	litter_saprotroph		1,2	-	-0.47	0.005	-0.49	0.003
p_Ascomycota,c_Leotiomycetes,o_Helotiales,f_Helotiales_fam_Incertae_sedis,g_*Mycoarthris*	litter_saprotroph		1	-	-0.33	0.052	-0.35	0.055
p_Ascomycota,c_Sordariomycetes,o_Hypocreales,_,_	N/A		1	+	0.33	0.042	-0.04	0.824
p_Ascomycota,c_Sordariomycetes,o_Hypocreales,f_Clavicipitaceae,g_*Metacordyceps*	animal_parasite	animal_decomposer	1	-	-0.35	0.056	-0.39	0.036
p_Ascomycota,c_Sordariomycetes,o_Hypocreales,f_Hypocreales_fam_Incertae_sedis,g_*Myxocephala* [2]	soil_saprotroph		2	-	-0.32	0.072	-0.35	0.047
p_Ascomycota,c_Sordariomycetes,o_Hypocreales,f_Nectriaceae,g_*Fusarium*	plant_pathogen	litter_saprotroph	1	-	-0.43	0.006	-0.42	0.009
p_Ascomycota,c_Sordariomycetes,o_Hypocreales,f_Nectriaceae,g_*Gibberella*	plant_pathogen	litter_saprotroph	2	-	-0.39	0.014	-0.35	0.029
p_Ascomycota,c_Sordariomycetes,o_Hypocreales,f_Nectriaceae,g_*Neonectria* [3]	plant_pathogen		1	+	0.33	0.044	0.21	0.197
p_Ascomycota,c_Sordariomycetes,o_Sordariales,f_Chaetomiaceae,_	N/A		1	-	-0.12	0.459	-0.32	0.047
p_Ascomycota,c_Sordariomycetes,o_Sordariales,f_Chaetomiaceae,g_*Humicola*	wood_saprotroph		1	-	-0.33	0.046	-0.44	0.005
p_Ascomycota,c_Sordariomycetes,o_Sordariales,f_Lasiosphaeriaceae,g_*Podospora*	dung_saprotroph	foliar_endophyte	1	+	0.39	0.018	0.28	0.086
p_Ascomycota,c_Sordariomycetes,o_Sordariales,f_Sordariales_fam_Incertae_sedis,g_*Staphylotrichum*	soil_saprotroph		2	+	0.39	0.015	0.27	0.100
p_Basidiomycota,c_Tremellomycetes,o_Cystofilobasidiales,f_Cystofilobasidiaceae,g_*Cystofilobasidium* [1]	litter_saprotroph		1	+	0.46	0.005	0.47	0.005
p_Basidiomycota,c_Tremellomycetes,o_Cystofilobasidiales,f_Mrakiaceae,g_*Mrakia*	saprotroph		1	+	0.39	0.017	0.30	0.066
p_Basidiomycota,c_Tremellomycetes,o_Filobasidiales,f_Piskurozymaceae,g_*Solicoccozyma*	soil_saprotroph	epiphyte	1	-	-0.10	0.558	-0.32	0.043
**p_Mortierellomycota,c_Mortierellomycetes,o_Mortierellales,f_Mortierellaceae,g_*Mortierella***	soil_saprotroph	root-associated	1	-	**-0.51**	**0.001**	**-0.49**	**0.002**
p_Olpidiomycota,c_Olpidiomycetes,o_Olpidiales,f_Olpidiaceae,_ [3]	N/A		1	+	0.48	0.004	0.38	0.027
p_Olpidiomycota,c_Olpidiomycetes,o_Olpidiales,f_Olpidiaceae,g_*Olpidium*	algal_parasite	plant_pathogen	2	-	-0.34	0.039	-0.35	0.029
Unidentified Fungi	N/A		1	-	-0.27	0.099	-0.32	0.047

Taxonomic levels shown as: p_: phylum, c_: class, o_: order, f_: family, g_: genus.

Bold: BH-corrected P <0.10. Otherwise, P<0.05.

Numbers next to taxa indicate they were also detected by other analyses: [1] *aldex.kw*, [2] indicator species, [3] both.

No taxa were detected only by *aldex.kw*

Table S7. Soil prokaryotic and fungal taxa (genus level) sensitive to cover crop (CC) treatments* which were also positively or negatively related to early crop growth. The table shows correlation coefficients with early crop growth, if correlations were also found at the phylum level, and which analysis detected CC effects.

Corr.	D/K	Phylum	Class	Order	Family	Genus	Pearson	Spearman	Phylum level?	Cc test
Positive	**Archaea**	**Crenarchaeota**	**Nitrososphaeria**	**Nitrososphaerales**	**Nitrososphaeraceae**	***Cand. Nitrocosmicus***	**0.35**	**0.48**	**yes**	corr1,2
	**Archaea**	**Crenarchaeota**	**Nitrososphaeria**	**Nitrososphaerales**	**Nitrososphaeraceae**	**Nitrososphaeraceae**	**0.61**	**0.70**	**yes**	corr2
	Bacteria	Actinobacteriota	Acidimicrobiia	Microtrichales	Iamiaceae	*Iamia*	0.27	0.36	yes	corr1
	Bacteria	Actinobacteriota	Acidimicrobiia	Microtrichales	uncultured	uncultured	0.30	0.45	yes	corr2
	**Bacteria**	**Actinobacteriota**	**Actinobacteria**	**Propionibacteriales**	**Nocardioidaceae**	***Nocardioides***	**0.30**	**0.46**	**yes**	corr1,2
	Bacteria	Actinobacteriota	Actinobacteria	Streptomycetales	Streptomycetaceae	*Streptomyces*	0.30	0.39	yes	corr2
	**Bacteria**	**Actinobacteriota**	**Rubrobacteria**	**Rubrobacterales**	**Rubrobacteriaceae**	***Rubrobacter***	**0.38**	**0.50**	**yes**	corr2
	**Bacteria**	**Actinobacteriota**	**Thermoleophilia**	**Gaiellales**	**Gaiellaceae**	***Gaiella***	**0.47**	**0.54**	**yes**	corr2
	**Bacteria**	**Actinobacteriota**	**Thermoleophilia**	**Solirubrobacterales**	**67-14**	**67-14**	**0.53**	**0.58**	**yes**	corr2
	Bacteria	Bdellovibrionota	Oligoflexia	Oligoflexales	Oligoflexales	*Oligoflexus*	0.33	0.37		corr2
	**Bacteria**	**Chloroflexi**	**Chloroflexia**	**Thermomicrobiales**	**JG30-KF-CM45**	**JG30-KF-CM45**	**0.51**	**0.58**		corr2
	Bacteria	Firmicutes	Bacilli	Alicyclobacillales	Alicyclobacillaceae	*Tumebacillus*	0.30	0.39	yes	indicsp;corr1,2
	Bacteria	Firmicutes	Bacilli	Bacillales	Bacillaceae	*Bacillus*	0.25	0.35	yes	corr2
	**Bacteria**	**Myxococcota**	**bacteriap25**	**bacteriap25**	**bacteriap25**	**bacteriap25**	**0.41**	**0.56**	**yes**	corr2
	**Bacteria**	**Proteobacteria**	**Alphaproteobacteria**	**Azospirillales**	**uncultured**	**uncultured**	**0.37**	**0.46**		corr2
	Bacteria	Proteobacteria	Alphaproteobacteria	Rhizobiales	Methyloligellaceae	uncultured	0.39	0.39		indicsp;corr2
Negative	Archaea	Crenarchaeota	Nitrososphaeria	Nitrosopumilales	Nitrosopumilaceae	*Cand. Nitrosotenuis*	-0.46	-0.44	yes	corr2
	Bacteria	Acidobacteriota	Acidobacteriae	Acidobacteriales	uncultured	uncultured	-0.21	-0.37		corr2
	**Bacteria**	**Acidobacteriota**	**Acidobacteriae**	**Subgroup_2**	**Subgroup_2**	**Subgroup_2**	**-0.38**	**-0.50**		corr2
	Bacteria	Acidobacteriota	Blastocatellia	Pyrinomonadales	Pyrinomonadaceae	RB41	-0.31	-0.32		corr2
	Bacteria	Acidobacteriota	Holophagae	Subgroup_7	Subgroup_7	Subgroup_7	-0.29	-0.33		corr2
	Bacteria	Actinobacteriota	Actinobacteria	Streptosporangiales	Thermomonosporaceae	*Actinocorallia*	-0.32	-0.40	yes	corr1
	Bacteria	Armatimonadota	Fimbriimonadia	Fimbriimonadales	Fimbriimonadaceae	Fimbriimonadaceae	-0.37	-0.34		corr2
	Bacteria	Bacteroidota	Bacteroidia	Cytophagales	Microscillaceae	uncultured	-0.42	-0.36		corr2
	**Bacteria**	**Bacteroidota**	**Kryptonia**	**Kryptoniales**	**BSV26**	**BSV26**	**-0.56**	**-0.49**		corr2
	**Bacteria**	**Chloroflexi**	**AD3**	**AD3**	**AD3**	**AD3**	**-0.34**	**-0.52**		indicsp;corr2
	Bacteria	Chloroflexi	Anaerolineae	Anaerolineales	Anaerolineaceae	uncultured	-0.32	-0.39		corr2
	Bacteria	Chloroflexi	Anaerolineae	SBR1031	A4b	A4b	-0.39	-0.36		corr1,2
	Bacteria	Desulfobacterota	Desulfuromonadia	PB19	PB19	PB19	-0.32	-0.34		indicsp;corr1,2
	Bacteria	Elusimicrobiota	Lineage_IIa	Lineage_IIa	Lineage_IIa	Lineage_IIa	-0.28	-0.41	yes	corr2
	Bacteria	Gemmatimonadota	Gemmatimonadetes	Gemmatimonadales	Gemmatimonadaceae	uncultured	-0.40	-0.41		corr2
	Bacteria	Proteobacteria	Alphaproteobacteria	Rhizobiales	Amb-16S-1323	Amb-16S-1323	-0.41	-0.37		corr1
	Bacteria	Proteobacteria	Gammaproteobacteria	Burkholderiales	Nitrosomonadaceae	MND1	-0.40	-0.40		corr2
	Bacteria	Spirochaetota	Spirochaetia	Spirochaetales	Spirochaetaceae	*Spirochaeta*	-0.34	-0.41	yes	corr2
	Bacteria	Verrucomicrobiota	Omnitrophia	Omnitrophales	Omnitrophaceae	*Cand. Omnitrophus*	-0.15	-0.37		corr1,2
	Bacteria	Zixibacteria	Zixibacteria	Zixibacteria	Zixibacteria	Zixibacteria	-0.35	-0.40	yes	indicsp;corr1,2

Fungi	Ascomycota	Sordariomycetes	Hypocreales	Nectriaceae	*Fusarium*	-0.37	-0.40	yes	corr1
Fungi	Ascomycota	Sordariomycetes	Hypocreales	Nectriaceae	*Gibberella*	-0.27	-0.29	yes	corr2
Fungi	Ascomycota	Sordariomycetes	Sordariales	Chaetomiaceae	*Humicola*	-0.34	-0.32	yes	corr1
Fungi	Ascomycota	Leotiomycetes	Helotiales	Helotiales_fam_Is	*Collembolispora*	-0.14	-0.33	yes	indicsp;corr1,2
Fungi	Mortierellomycota	Mortierellomycetes	Mortierellales	Mortierellaceae	*Mortierella*	-0.36	-0.38	yes	corr1

* Sensitivity to CCs was evaluated with three analyses: indicator species (indicsp; Tables 1 and 2) or *aldex.corr* with CAP1 and CAP2 (corr1 and corr2, respectively; Figs. 2a and 2d. These taxa, and those correlated with early crop growth, were selected using a flexible criterion (P<0.05) for a broader exploration. Taxa whose correlation with crop growth also had a Benjamini-Hochberg-corrected P<0.10 are shown in **bold**.

No fungal genera were positively correlated with early crop growth.

D/K: Domain/Kingdom, Cand.: *Candidatus*

Table S8. Mean values of soil physicochemical properties across cover crop (CC) and residue management treatments.

	R-										R+									
	no-CC		oat		radish		rye		rye-radish		no-CC		oat		radish		rye		rye-radish	
N-NO_3^- (ppm)	9.18	bc	9.00	c	16.19	a	8.33	c	10.56	b	9.65	b	8.60	b	16.46	a	9.14	b	9.72	b
N-NH_4^+ (ppm)	3.90	a	3.63	ab	6.25	a	2.87	b	4.37	a	4.49	a	3.75	a	5.37	a	3.99	a	3.79	a
SOC (%)	2.87	a	3.07	a	3.03	a	2.90	a	3.05	a	2.95	a	2.99	a	3.06	a	3.03	a	3.06	a
Total N (%)	0.28	b	0.31	a	0.32	a	0.33	a	0.32	a	0.28	b	0.32	a	0.33	a	0.32	a	0.33	a
C:N	11.93	a	11.10	a	10.20	a	10.28	a	11.10	a	11.60	a	11.78	a	10.60	a	10.43	a	10.68	a
Sand (%)	77.25	a	76.05	a	77.20	a	76.25	a	75.20	a	76.83	a	76.30	a	76.05	a	76.23	a	76.40	a
Silt (%)	17.20	a	19.10	a	18.48	a	18.53	a	19.30	a	18.43	a	18.83	a	19.05	a	19.15	a	17.43	a
Clay (%)	5.55	a	4.83	a	4.28	a	5.23	a	5.53	a	4.75	a	4.85	a	4.95	a	4.63	a	6.18	a

Nitrate, ammonium, soil organic carbon (SOC), total N and C:N ratio measured in April 2016 (before tillage). Texture measured in September 2016 (at tomato harvest).
Different letters show significant differences between CC treatments, within each residue management treatment (Tukey test, alpha=0.05).
No significant residue management effects were observed.
These data and measurements for other dates are available in previous publications (Chahal and Van Eerd 2018, 2019, 2020).

Table S9. Main crop sequence, residue removal and fall planting in the studied mid-term trial from Ridgetown, ON. Sampling year is shaded in grey.

Year	Main crop	Residue removal	Fall planting
2008	Processing pea (*Pisum sativum* L.)	No	Cover crops
2009	Processing sweet corn (*Zea mays* L. var. *saccharata*)	No	Cover crops
2010	Spring wheat (*Triticum aestivum* L.)	No	Cover crops
2011	Processing tomato (*Solanum lycopersicum* L.)	No	Cover crops
2012	Grain corn (*Zea mays* L.)	Yes	None
2013	Processing squash (*Cucurbita pepo*)	No	Cover crops
2014	Soybean (*Glycine max* L.)	No	Winter wheat
2015	Winter wheat (*Triticum aestivum* L.)	Yes	Cover crops
2016	Processing tomato (*Solanum lycopersicum* L.)	No	Cover crops

Table S10. Agricultural management details in the studied mid-term trial from Ridgetown, ON.

			Pea (2008)		Sweet corn (2009)		Wheat (2010)		Tomato (2011)		Grain corn (2012)		Squash (2013)		Soybean (2014)		Winter wheat (2015)		Tomato (2016)	
	Activity		Date	Details	Date	Details	Date	Details	Date	Details	Date	Details	Date	Details	Date	Details	Date	Details	date	details
Cc	Herbicide termination				06-May	glyphosate 1853 gr/ha	20-Apr	glyphosate 1853 gr/ha	06-May	glyphosate 1853 gr/ha			02-May	glyphosate 1333 gr/ha	13-May	glyphosate 1333 gr/ha			03-May	glyphosate 1333 gr/ha
Main	Mowing	flail mower to 10 cm above ground			21-May	stock chopped							27-May	stock chop stubble	22-May	stock chopped				
	Fertilizer	4Rs for N,P,K	23-Apr	0-23-30, 280 kg/ha	29-May	0-23-30, 292 kg/ha	05-May	0-23-30, 449 kg/ha	30-May	0-23-30, 449 kg/ha	17-May	0-23-30, 336 kg/ha	24-May	0-23-30, 336 kg/ha					24-May	0-23-30, 336 kg/ha
	Tillage	implement and #times			21-May	disked x2, cultivated	04-May	disked	25-May	disked and cultivated			31-May	disk x2 and cultivate					24-May	disked and cultivated x2
	(Trans)Planting	population	23-Apr	drilled 280 kg/ha of pea variety spring	02-Jun	air seeder, 54,587/ha	05-May	drilled hobson, 3.458 mil/ha	31-May	transplanted TSH18 39,000/ha	18-May	variety 52-59, 79,040 seeds/ha	31-May	autumn delight air seeder 5808 seeds/ac		sometime between may22 and jun12	oct 23 2014	drilled, 25R40 3.458 mil/ac	25-May	transplanted 1178's, 12,400 plants/ac
	Insecticide	a.i. and rate							31-May	imidacloprid 14.82 gr/ha										
	Herbicide	a.i. and rate	24-Apr	s-metolachlor 1582 gr/ha	03-Jun	s-metolachlor 1356 gr/ha	31-May	bromoxynil/MCPA 280 gr/ha	30-May	s-metolachlor 1582 gr/ha	25-May	s-metolachlor 1130 gr/ha	05-Jun	s-metolachlor 1039 gr/ha	17-Jun	glyphosate 1333 gr/ha	25-Jun	bromoxynil/MCPA 280 gr/ha	24-May	s-metolachlor 1582 gr/ha
	Herbicide	a.i. and rate	13-May	sethoxydim 500gr/ha	03-Jun	bentazon 830 gr/ha			30-May	metribuzin 555 gr/ha					11-Jul	glyphosate 1333 gr/ha			13-Jun	metribuzin 177 gr/ha
	Herbicide	a.i. and rate	29-May	MCPB/MCPA 1700 gr/ha																
	herbicide	a.i. and rate	05-Jun	bentazon 830 gr/ha																
	Fertilizer	4Rs for N if applicable	23-Apr	247 kg/ha of 27-0-0			01-Jun	46-0-0, 171 kg/ha	30-May	46-0-0, 140 kg/ha	11-Jun	UAN, 123.5gal/ha	03-Jun	27-0-0 407 kg/ha			27-Apr	46-0-0 247 kg/Ha		
	Insecticide	a.i. and rate											20-Jun	imidacloprid 118 gr/ha						
	Fungicide	a.i. and rate							20-Jun	chlorothalonil 1852.5 gr/ha			20-Jun	cyazofamid 49.4 gr/ha					13-Jun	chlorothalonil 1852.5 gr/ha
	Insecticide	a.i. and rate											26-Jun	imidacloprid 118 gr/ha						
	Fungicide	a.i. and rate							30-Jun	copper hydroxide 24.4% 2.47 L/ha			26-Jun	propamocarb/chlorothalonil 926 gr/ha					06-Jul	chlorothalonil 1852.5 gr/ha
	Insecticide	a.i. and rate											09-Jul	imidacloprid 118 gr/ha						
	Fungicide	a.i. and rate							15-Jul	chlorothalonil 1852.5 gr/ha			09-Jul	cyazofamid 69.2 gr/ha						
	Fungicide	a.i. and rate							02-Aug	chlorothalonil 1852.5 gr/ha			09-Jul	chlorothalonil 2469 gr/ha					02-Aug	chlorothalonil 1852.5 gr/ha
	Fungicide	a.i. and rate							10-Aug	chlorothalonil 1852.5 gr/ha			29-Jul	cyazofamid 69.2 gr/ha						
	Fungicide	a.i. and rate							10-Aug	mandipropamid 230 ml/ha			29-Jul	chlorothalonil 2469 gr/ha						
	Harvest	hand or combine	27-Jun	hand harvest	25-Aug	hand harvest	12-Aug	plot combine	29-Aug	hand harvest	24-Oct	machine harvest	27-Aug	hand harvest	22-Oct	plot combine harvest	22-Jul	plot combine	06-Sep	hand harvest
	Herbicide	a.i. and rate	14-Jul	glyphosate 1853 gr/ha							24-Oct	stock chopped								
	Mowing	flail mower to 10 cm above ground	02-Jul	stock chopped	31-Aug	stock chopped	23-Aug	stock chopped	15-Sep	stock chopped										
Cc	Tillage	implement and #times			01-Sep	disked	07-Sep	disked x2 and cultivated	15-Sep	disked and cultivated			10-Sep	soil saved and cultivated			12-Aug	disked	21-Sep	disk ripped, cultivated
	Planting	drill	22-Jul	planted cover crops with drill	03-Sep	planted cover crops with drill	08-Sep	drilled covercrops	16 set	planted covercrops with drill			11-Sep	planted covercrops with drill	23-Oct	drill wheat, 25R40 3.458 mil/ha	17-Aug	planted with drill	22-Sep	planted with drill
	Herbicide (control plots)	a.i. and rate									06-Nov	glyphosate 1333 gr/ha					10-Sep	glyphosate 1333 gr/ha, no cover plots		
